# The impact of interactions on invasion and colonization resistance in microbial communities

**DOI:** 10.1371/journal.pcbi.1008643

**Published:** 2021-01-22

**Authors:** Helen M. Kurkjian, M. Javad Akbari, Babak Momeni

**Affiliations:** Department of Biology, Boston College, Chestnut Hill, Massachusetts, United States of America; EMBL-Heidelberg, GERMANY

## Abstract

In human microbiota, the prevention or promotion of invasions can be crucial to human health. Invasion outcomes, in turn, are impacted by the composition of resident communities and interactions of resident members with the invader. Here we study how interactions influence invasion outcomes in microbial communities, when interactions are primarily mediated by chemicals that are released into or consumed from the environment. We use a previously developed dynamic model which explicitly includes species abundances and the concentrations of chemicals that mediate species interaction. Using this model, we assessed how species interactions impact invasion by simulating a new species being introduced into an existing resident community. We classified invasion outcomes as resistance, augmentation, displacement, or disruption depending on whether the richness of the resident community was maintained or decreased and whether the invader was maintained in the community or went extinct. We found that as the number of invaders introduced into the resident community increased, disruption rather than augmentation became more prevalent. With more facilitation of the invader by the resident community, resistance outcomes were replaced by displacement and augmentation. By contrast, with more facilitation among residents, displacement outcomes shifted to resistance. When facilitation of the resident community by the invader was eliminated, the majority of augmentation outcomes turned into displacement, while when inhibition of residents by invaders was eliminated, invasion outcomes were largely unaffected. Our results suggest that a better understanding of interactions within resident communities and between residents and invaders is crucial to predicting the success of invasions into microbial communities.

## Author summary

Our resident microbiota can prevent diseases by making it harder for pathogens to grow and establish, a phenomenon called “colonization resistance.” Colonization resistance is one of the major benefits provided by human-associated microbiota and a viable alternative to the use of antibiotics for preventing or treating infections. Here we use a model of microbial interactions through production and consumption of metabolic compounds to assay invasion and colonization resistance. We systematically examine in simulations how interactions among resident members and those between residents and an invader impact colonization resistance and invasion outcomes. In our simulations, the common strategy of increasing the dosage of probiotics is often unsuccessful for augmenting a new species into a resident microbiota. Instead, we find that the net facilitation or inhibition between the resident members and the invader explains whether the community remains intact and whether the invader can establish. Our results suggest that a better understanding of microbial interactions can inform successful microbiota interventions.

## Introduction

Members of resident communities can influence whether invading species are able to establish in an ecosystem. In human microbiota, where invasion is a first step in the establishment of many pathogens, preventing the invasion is sometimes referred to as colonization resistance. The potential for resident microbes to protect us from pathogens has been observed as early as 1917, by the discovery of *Escherichia coli* Nissle 1917 that antagonized and blocked enteric pathogens [1]. More examples across different microbiota sites abound: nasal microbiota can protect us against respiratory *Staphylococcus aureus* infection [2,3], gut microbiota can protect from *Clostridium difficile* infection [4,5], and oral microbiota can block *E*. *coli* infection [6], to name a few. While substantial evidence demonstrates that resident microbiota can suppress pathogens [7–9], the mechanism of colonization resistance is not fully understood [6].

Strategies to promote the addition of new taxa to human microbiota—called engraftment—can also be critical components of healthcare. For example, fecal microbiota transplant is used to increase the diversity of human gut microbiota and treat a variety of intestinal conditions, including *C*. *difficile* infection [10], Crohn’s disease [11], and ulcerative colitis [12]. The application of probiotics has become an important component of current approaches for promoting a healthy neonatal gut microbiome [13–15] as well as for treating bacterial vaginosis [16] and numerous digestive disorders [17]. Additional exploration of the community and environmental contexts in which each strategy is likely to be effective could benefit from guidance from appropriate theory. However, with a few exceptions (such as [6,18,19]), and despite the many studies on invasion and colonization resistance performed in microbial systems [20–23], the potential repercussions of mechanisms of colonization resistance and invasion ecology on microbiota intervention strategies have not been explored in depth.

Resident communities can affect invasion outcomes by altering resource availability, occupying niches, or interacting with invaders directly or indirectly via predation, competition, facilitation, or other mechanisms. The relative importance of factors determining invasion outcomes varies across communities and ecosystems. Functional composition of resident communities, for example, is a major determinant of invasion success in many grasslands [24], while release from consumer or competitive pressure is an especially important factor in marine invasions [25]. The majority of explanations about how microbiota achieve or resist colonization are built around competition [26–29], which can take many forms [30,31], including resource competition, production of toxic compounds, or induction of host immunity. The role of facilitation—interactions that benefit other community members—has been less frequently investigated, despite being considered an important influence on invasion outcomes in non-microbial systems for decades [32–38]. To highlight a few examples, it has been shown that incidental mutual interactions with native species promotes invasion [39], facilitation among two non-native species can make invasion more successful [40], and facilitative ecosystem-engineering can increase invasion success [37]. Among microbes, interactions often occur via chemical mediators released into the environment [41,42] and are believed to be influential in structuring many microbial communities. The impact of positive interactions on invasion outcomes, especially in the context of microbes interacting via diffusing chemical mediators, remains underexplored.

Mathematical models have been used to explore many aspects of the relationship between interspecific interactions (chemically-mediated and otherwise), microbial community structure, and invasion fate. Modeling of metabolic interaction networks have provided insight into how nutrient exchange can lead to emergent properties of microbial communities, such as biodegradation or the ability to survive in nutrient poor environments [43]. And by modeling the microbes of the human colon as functional groups producing a range of major metabolic products, Kettle and colleagues were able to reproduce experimental results showing changes in community composition in response to pH change [44]. Modeling of invasion of a new species into a multispecies biofilm has shown that colonizing microbes can move from bulk liquid into the biofilm and establish where environmental conditions favor their growth [45,46]. Models of bacteriocin-mediated interactions have demonstrated that Lactic Acid Bacteria could be effectively used to control *Listeria sp*. growth in food products [47,48]. However, none of these models have explored the conditions under which positive and negative chemically-mediated interactions within resident communities and between residents and invaders might alter invasion outcomes.

Mechanistic studies typically fall along a spectrum of generality. In invasion ecology, on one side are general theoretical predictions [29,35,38], while on the other side are specific, although remarkable, instances such as host-supported colonization of legume by rhizobia [49,50]. Studies in human microbiota can be done in these two types as well (see a summary of different model systems in [51], for example). Here, we choose to focus on developing general insights that could inform and guide future microbiota-based intervention strategies. Since such general insights are hard to draw in natural microbiota, where members and interactions are not often adequately known, we use *in silico* models in our approach instead.

We use a previously introduced mathematical model of microbes and explicit mediators of interactions [52,53] to investigate how invasion of microbial communities is affected by chemical-mediated interspecific interactions between the invader and resident members or among resident members. Even though the formulation of our model is inferred from empirical data in [53], our conclusions are derived from many constructed *in silico* examples; as such, they do not reflect specific metabolites and are insensitive to the details of the parameters used. As described below, our investigation suggests that species interactions can markedly influence invasion and colonization resistance.

## Results

### Increasing the propagule size does not increase the chance of incorporation of an invader into a resident community

**Fig 1:**
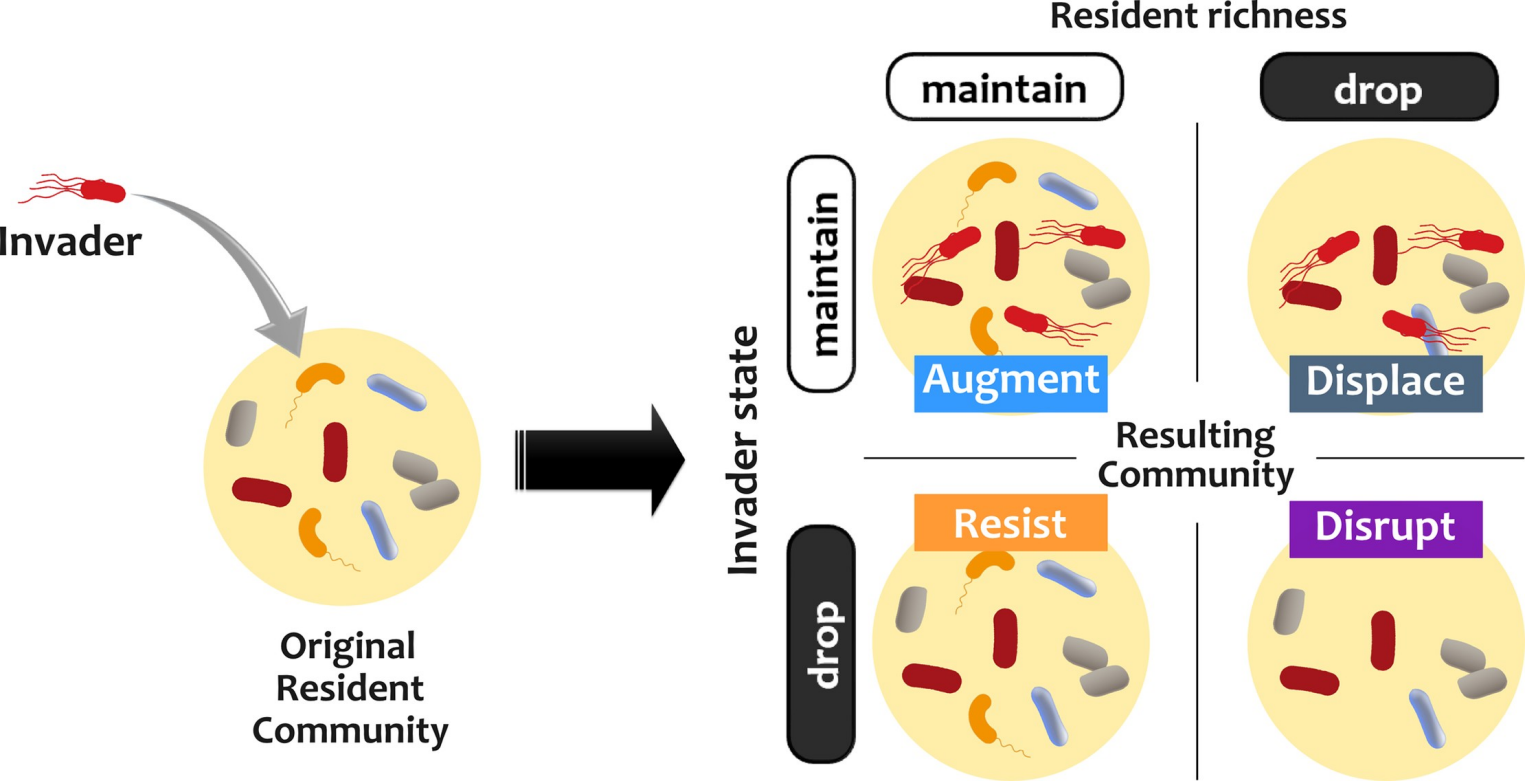
An *in silico* invasion assay allows us to assess invasion outcomes. In our *in silico* invasion assay, we first assemble instances of stable resident communities and then assess the outcomes after introducing an invader. Based on whether the invader persists or goes extinct and whether the resident community maintains or decreases in richness, we categorize the outcomes into four groups: ‘Resistance’ (invader extinct, resident richness maintained), ‘Augmentation’ (invader maintained, resident richness maintained), ‘Disruption’ (invader extinct, resident richness drops), and ‘Displacement’ (invader maintained, resident richness drops).

To assess colonization resistance, we set up an *in silico* invasion assay in which stable resident communities [54] are challenged with invaders introduced at different population sizes (Fig 1; see Materials and Methods for details). We categorize the outcome based on the fate of the invader and the resident community. There are four possible outcomes (Fig 1): ‘Resistance’ (invader extinct, all resident species maintained), ‘Augmentation’ (invader maintained, all resident species maintained), ‘Disruption’ (invader extinct and some resident species also go extinct), and ‘Displacement’ (invader maintained and some resident species go extinct).

**Fig 2:**
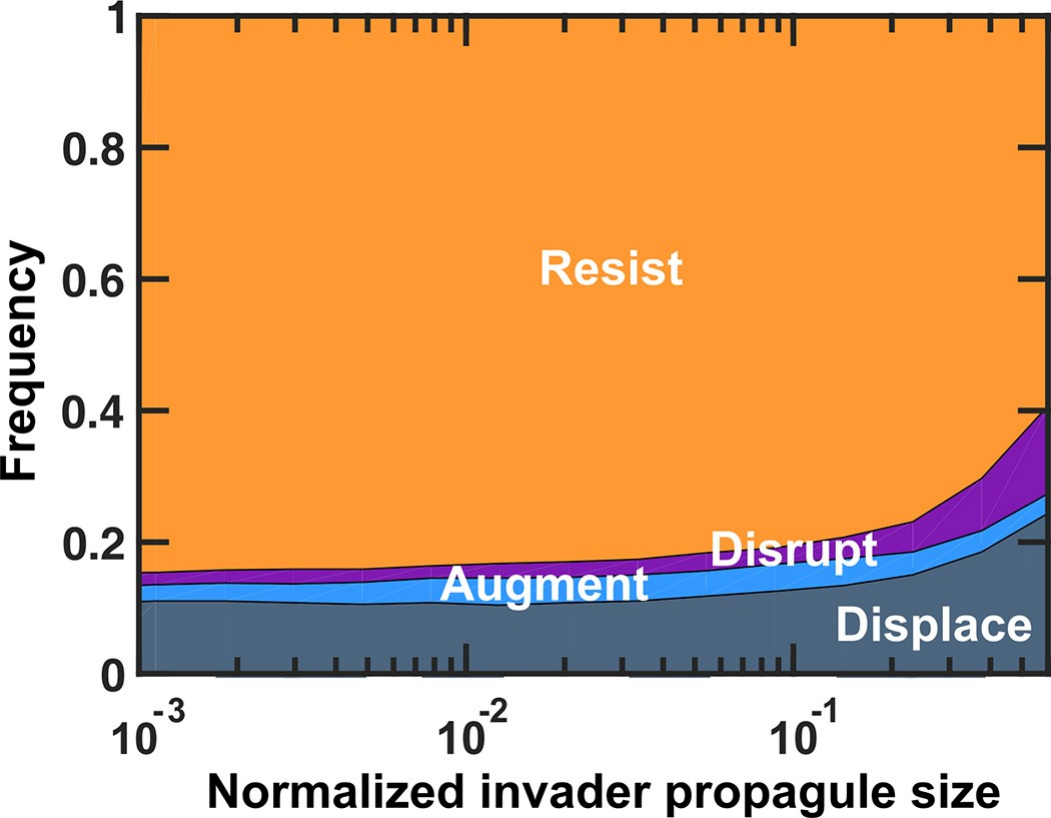
Only at large invader propagules colonization resistance is weakened. As the normalized propagule size (*i*.*e*. the amount of invader cells introduced, relative to the total population size of the resident community) increases, the probability of resistance decreases, the probability of disruption and displacement increases, and the probability of augmentation remains approximately constant. Number of instances examined N_s_ = 1000. Interactions among resident members are equally likely to be facilitative or inhibitory (*f*_*fac*_ *=* 0.5). Interactions between resident members and the invader are mostly inhibitory (*f*_*fac*,*inv*_ = 0.1). The invader has on average a 50% advantage in basal growth rate compared to resident members (*r*_*0*,*inv*_/*r*_*0*,*res*_ = 1.5). To visualize the trends more clearly, here we do not include the error-bars (see S1 Fig for confidence intervals).

We observe that only when the relative size of the invader population introduced into the resident community—hereafter called propagule size—is comparable to the resident community, the chance of observing different outcomes is affected (Figs 2 and S1). In our model, no considerable change in outcomes is observed below a propagule size of ~10%. Community outcomes are affected when invader propagule size exceeds ~10% of the resident population. Probability of resistance decreases across this range. Importantly, in our model, a larger propagule size does not appear to alter the chance of augmentation. At large propagule sizes, the invader is maintained in the final population only at the cost of losing some of the resident species. This is at odds with common wisdom of using probiotic at high doses to allow the “helpful” microbes to be augmented into the microbiota.

### Invaders with higher basal growth rate are more likely to displace residents

We asked if the basal growth of the invader—what would conventionally be the main indicator of its competitive potential—is the major determinant of invasion success. To answer this question, we introduced invaders with different basal growth rate—i.e. growth rate in the absence of interactions—into the community and tallied the invasion outcomes (S2 Fig). The results show that as the community is challenged with invaders with basal growth rates higher than resident members, the outcome shifts from resistance to displacement. The augmentation and disruption outcomes remain unlikely as the basal growth rate of the invader increases.

### Facilitation of invader by resident microbiota can weaken colonization resistance

**Fig 3:**
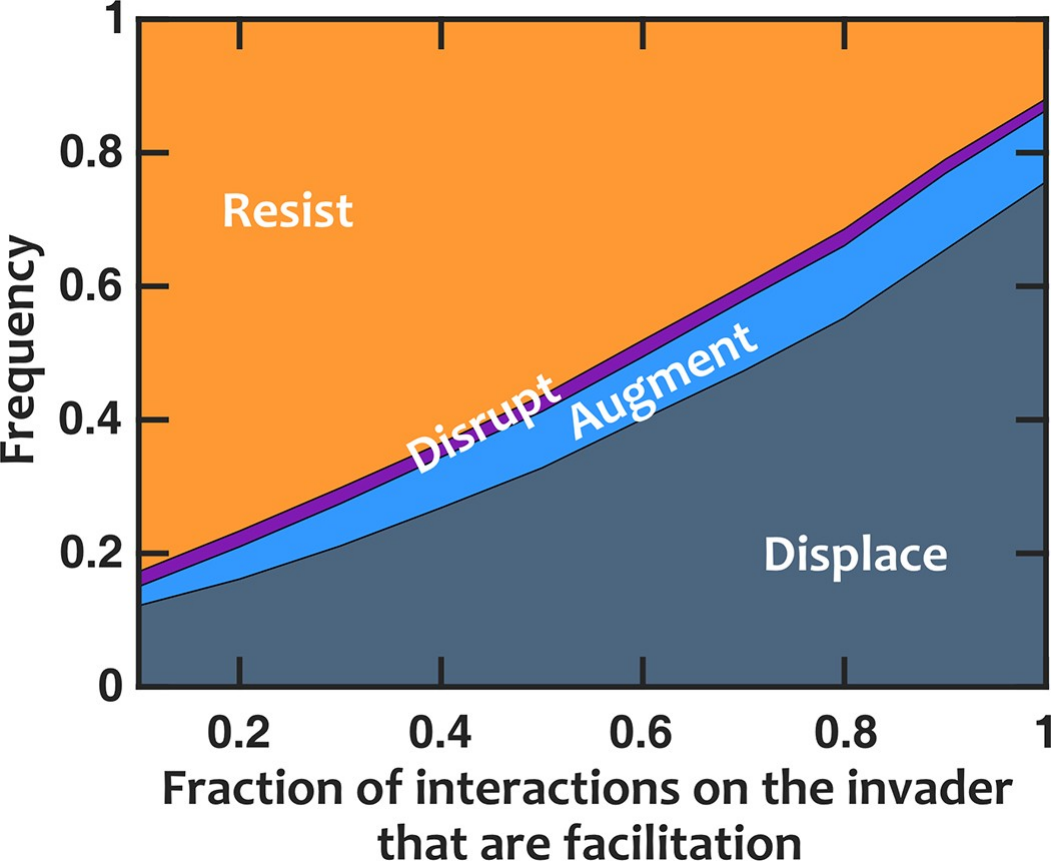
When resident species facilitate the invader, colonization resistance is weakened. Invasion success drastically increases when we switch the interactions that influence the invader from inhibition to facilitation. Number of instances examined N_s_ = 1000. Interactions among resident members are equally likely to be facilitative or inhibitory (*f*_*fac*_ *=* 0.5). Normalized basal growth rate of the invader is 1.5. Normalized introduced propagule size is 0.3%.

To investigate the impact of interactions on invasion outcomes, we first looked at the interactions between the resident microbiota and the invader. We kept the interactions within the resident communities fixed and transitioned the interactions imposed by the resident microbiota on the invader from mostly inhibition to mostly facilitation (Fig 3). The results show a clear trend: when the resident community facilitates the invader, the chance of invasion (augmentation or displacement outcomes) is enhanced. As facilitation of the invader increases, the fraction of resistance decreases, with a corresponding increase in displacement. The chance of augmentation increases slightly, while the probability of disruption remains relatively constant.

This result is fairly intuitive. If the resident members mostly facilitate the invader, the growth rate boost that the invader receives makes the invasion more successful. The prevalence of the displacement category in these results rather than augmentation (i.e. successful invasions tend to deplete resident community richness) reinforces the view that facilitation of invaders may be detrimental to resident communities.

### More cooperative microbiota show stronger colonization resistance

**Fig 4:**
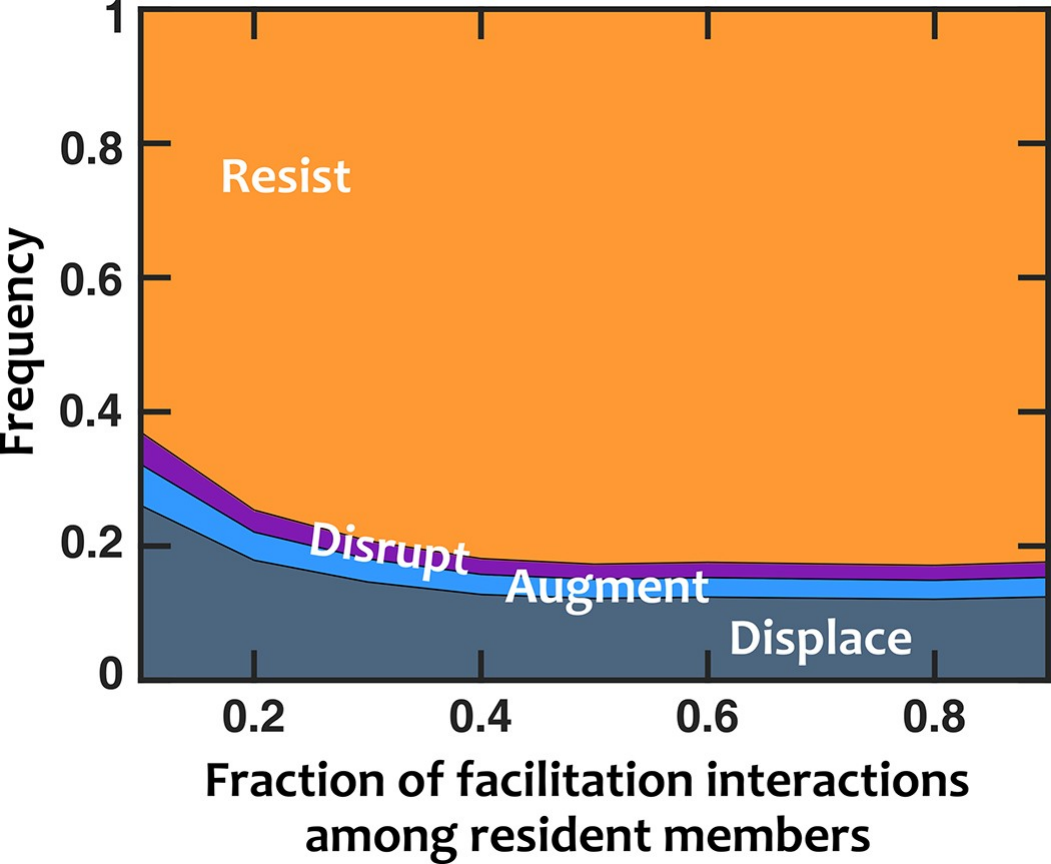
Facilitation among resident species strengthens colonization resistance. Invasion success decreases when interactions among resident species are predominantly facilitation rather than inhibition. The interactions between resident species and the invader are mostly inhibitory (*f*_*fac*,*inv*_ *=* 0.1). Normalized basal growth rate of the invader is 1.5 (compared to resident members). Normalized introduced propagule size is 0.3%.

We shifted our focus to the interactions within the resident microbiota to assess their impact on invasion outcomes. For this, we surveyed many examples of stable resident communities formed by groups of species that engaged in interactions at different facilitation to inhibition fractions. The results showed that invasion was less successful when there was prevalent facilitation among resident members (Fig 4). This trend holds when the resident members are mostly antagonistic against the invader as well (S3 Fig).

One explanation for the above results is from the perspective of available interactions. The mediators present in the resident community can modulate the growth rate of the invader positively or negatively. If other members of the community are already mostly positively benefitting from these mediators, that leaves less room for an invader to take advantage of those resources to keep up with resident members or outpace them in growth.

### The net interaction between the invader and resident microbiota determines the type and strength of colonization resistance

**Fig 5:**
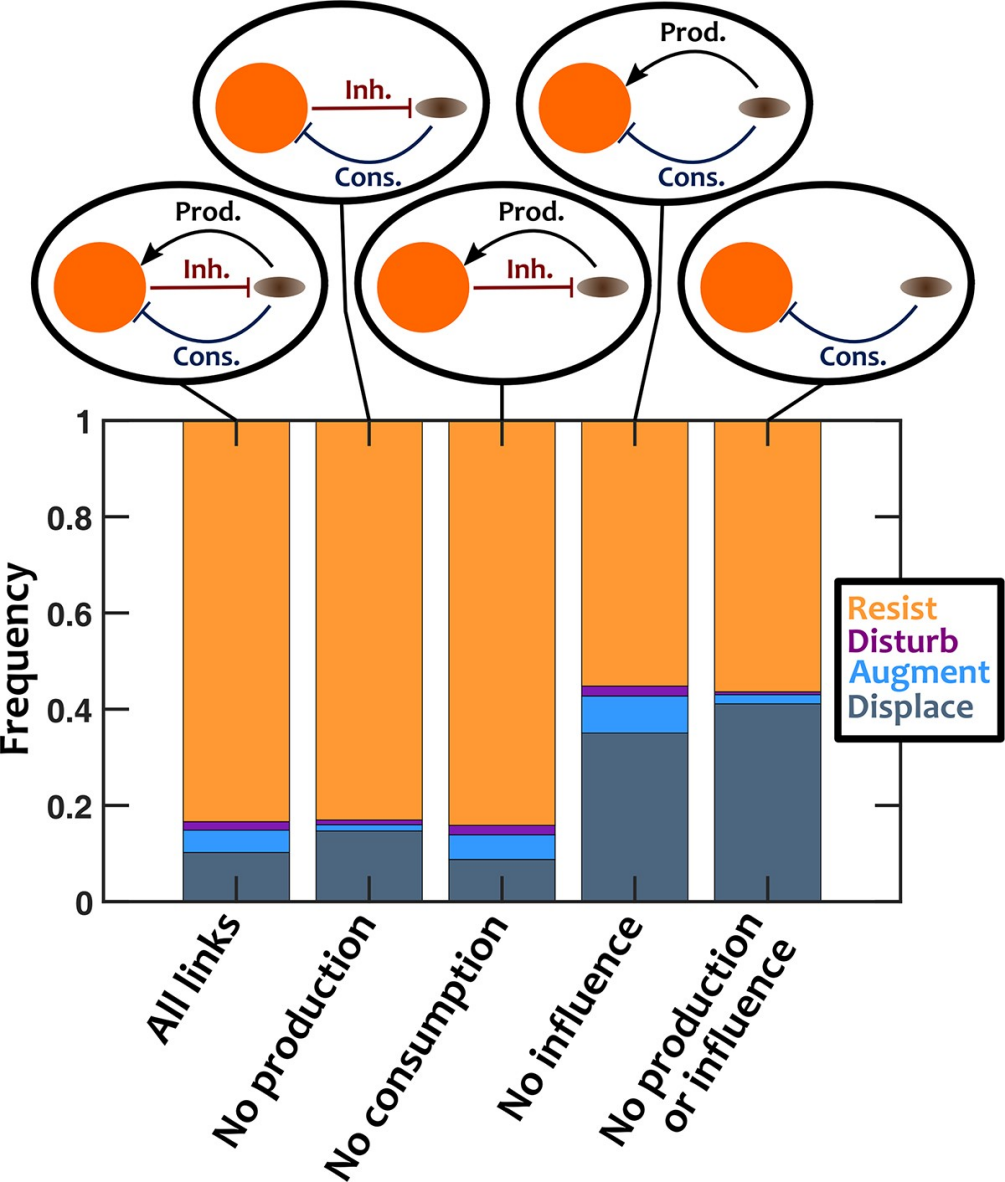
The effective interaction between the resident community and the invader alters the outcome of invasion. We examined how invasion outcomes changed when the interactions between the invader and resident members were altered. In the original case, the invader is inhibited by resident species, and the invader consumes and produces some chemical mediators. In other cases, the invader inhibition, consumption, or production (or a combination of them) are removed from the system, as shown in the insets, to assess the impact of each. Interactions among resident species are equally likely to be facilitative or inhibitory (*f*_*fac*_ *=* 0.5). The influence of residents on the invader is mostly inhibitory (*f*_*fac*,*inv*_ *=* 0.1). Invader has a normalized basal growth rate of 1.5. Number of instances examined N_s_ = 10000.

What are the mechanisms through which interactions between the invader and resident members determine the chance of invasion? We considered a particular case where the chemical mediators of the resident were more likely to be inhibitory to the invader. We asked how much this inhibitory effect as well as consumption or production of mediators by the invader influenced the invasion outcome. The invader interfaces with residents in two ways: it can affect the chemical environment of the resident community by consuming or producing chemical mediators, and it also gets affected by the chemicals in the environment. As expected, when more mediators had an influence on the invader, colonization resistance was strengthened (S4A Fig). We tested how removing production, consumption, and/or chemical influence on the invader impacted invasion outcomes (Figs 5 and S5).

To interpret the results, we first recall that the resident community involves mostly facilitative interactions among resident members [53]. Thus, we expect production of mediators by the invader to facilitate the resident community, while consumption of mediators will inhibit residents. Consistent with this expectation, when more of the mediators were produced by the invader, the chance of augmentation increased (S4B Fig) and depletion of mediators weakened colonization resistance (S6 Fig). We can simplify our view of the system as two compartments—*i*.*e*. the resident community and the invader—and their interactions. As shown in the left-most inset of Fig 5, in the original case the invader facilitates the resident community through production of mediators, inhibits the resident community through consumption of mediators, and gets inhibited by the chemical environment of the resident community (our assumption for this example). Examining the results after removing different components of this model leads to several interesting observations: (1) Removing invader production (thus, no facilitation) removes the augmentation types from the outcomes and increases the chance of displacement. (2) Removing consumption (thus, no inhibition of microbiota by the invader) only marginally decreases the chance of displacement and slightly improves colonization resistance. (3) Removing the inhibition of the invader increases the chance of invasion, primarily by contributing to the displacement outcomes. The results are overall in agreement with what we would expect from a two-component system [55]: facilitation increases stable coexistence (manifested as the augmentation outcome), whereas mutual inhibition increases bistability (most outcomes are resistance or displacement).

Insights from the impact of different interactions on invasion and colonization resistance outcomes (Figs 5 and S5) is a helpful first-step to rethink the intervention strategies for manipulating microbiota. Our results suggest that when interactions other than pure competition are involved, propagule size is not an effective parameter to influence the outcomes (Fig 2). Instead, interactions between the invader and the resident community and the interactions within the resident community appear to influence the outcome (Fig 5). Take a situation of assimilating a probiotic strain into an existing microbiota. Our results suggest that rather than administering larger doses of the probiotic strain, the focus should be on creating facilitation or removing existing inhibition between the probiotic strain and the resident microbiota.

## Discussion

To examine how interspecific interactions affect invasion outcomes within microbiota, we used a mediator-explicit model of chemical interactions among microbes [52,53]. We developed an *in silico* yet empirically feasible assay to assess different types of invasion. Invasion outcomes in this assay are categorized as resistance, augmentation, disruption, or displacement depending on whether the invader is maintained in the community or driven extinct and whether the resident community maintains its richness or some resident species go extinct. Using this model, we investigated the impact of different parameters—including those related to interactions—on invasion outcomes.

We saw that as the size of the invader population increased relative to the resident community, resistant outcomes decreased and were replaced by disruptions. Empirical work on invasion of colicinogenic bacteria into a colicin-sensitive population found that initial population size of the invader had no effect on invasion outcome, but did have a negative relationship with time to displacement of the resident community [56]. Other work in microbial communities has found that invasions into structured environments are only successful with a sufficiently large initial population size or propagule pressure [57–59]. We also found that when invaders had higher basal growth rates relative to the resident members, resident communities were increasingly disrupted, which agrees with empirical results from microbial systems showing a positive relationship between invader success and invader growth rate [60–62]. Other work has found no effect of invader growth rate on invasion outcome [56].

As expected, we found that invaders are more successful if the chemical environment created by the resident community facilitates them. We also observed that if the interactions among members that form the resident community are more facilitative—rather than inhibitory—colonization resistance is strengthened. This is in contrast to empirical work demonstrating that facilitative interactions promote invasion, including invasion of the Asian shore crab into intertidal communities [37] and the plant pathogen *Ralstonia solanacearum* into rhizosphere communities [63], although the promotion of invasion in the latter example was believed to be due to facilitation of the invader by facilitative resident communities, while in our work interactions among community members and between residents and invader were explicitly considered separately. In fact, facilitation has been proposed as a mechanism to explain the positive relationship between diversity and invasibility that is often observed at broad spatial scales [29,64]. Because space is not explicitly considered in our model, we cannot comment on the possibility that scale may account for the disparity between our results and those from empirical systems, however this could be an important area for future exploration. Finally, we found that when facilitation of the residents by the invader is removed, augmentation outcomes are replaced by displacement, while when inhibition of residents by invaders is removed, there is little change in invasion outcomes.

The tradeoff in prevalence between resistance and displacement outcomes—created by facilitative and inhibitory interspecific interactions, respectively—in our results already highlights how such interactions influence colonization resistance. This on its own is not a new finding. Many studies before us have pointed to the impact of interactions, positive or negative, on invasion, including [6,26,33,37], and microbial communities that are unable to resist invasion frequently decrease in resident diversity [65]. Our contribution is to highlight this impact in a model of continuous growth in microbiota in a framework based on consumption and production of chemicals that can impact cell growth and to track down how these processes lead to an eventual success or failure of an invader. It is striking to note the many different ways invasion trajectories can be altered by modifying or removing interspecific interactions. An increase of facilitation of the invader, for example, leads to an increase in displacement and augmentation, both outcomes in which the invader is maintained in the final community. By contrast, facilitation among residents increases colonization resistance, while the loss of facilitation of residents by the invader leads to a shift from augmentation, in which the full resident community richness is maintained, to displacement, in which one or more residents are driven extinct. The context dependence of the effects of positive interactions on invasion outcomes emphasizes the importance of improving our understanding of the natural history of microbial communities.

A simple two-compartment model of invader versus the resident community offers an intuitive prediction of invasion outcomes. If the effective interaction between the resident community and the invader is mutual inhibition, we would expect bistability—either the resident community resists invasion or it crumbles. In contrast, for assimilation of an invader into microbiota, a facilitative interaction between the resident members and the invader appears necessary. When interactions are the main driving force, two-way facilitation in a mutualism or one-way facilitation in a commensalism or prey-predation interaction are necessary for stable coexistence of the two components [55].

This work was conducted using a model in which microbes interact exclusively via chemical mediators released into the environment. Such interactions are common in microbial communities and are important determinants of community assembly and other processes, but they are by no means the only important microbial interactions. For example, in many microbiota, contact dependent growth inhibition [66] and type VI secretion systems [67] are critical mechanisms of bacterial competition that rely on contact between interacting cells. Many interactions are also mediated by induction of the host immune system [68]. This work cannot account for the possible roles of such interactions influencing invasion trajectories or predict the relative importance of chemical-mediated interactions on invasion outcomes. Direct comparison of the impact of these alternative interaction mechanisms on invasion outcomes would provide an important extension of this work.

In our model, we intentionally have focused on interspecies interactions beyond competition for resources. An important question is whether the trends we have found will hold when resource competition is included in the model. To answer this question, we modified our model and explicitly incorporated competition for a single limiting resource (see Materials and Methods). We chose the amount of resource such that within each round of growth the populations would deplete the resource, ensuring that resource competition was in effect. We found that making the resource limitation more stringent did not considerably influence the outcome of invasion (S7 Fig). Our results show that the low sensitivity of outcomes to the propagule size still holds in this modified model (S8 Fig). Changing the basal growth rate of the invader also resulted in an overall trend resembling the case with no explicit resource competition, with only one qualitative difference: an increased occurrence of disruption at intermediate levels of invader growth rate (S9 Fig). The trends of invasion outcomes based on invader-resident interactions (S10 Fig) and resident-resident interactions (S11 Fig) also largely remained intact.

Finally, by helping us to understand under what circumstances particular invasion outcomes are likely, this work can help to guide interventions towards those that are appropriate for restructuring microbiota. For example, the common strategy of increasing the dosage of probiotics to increase the chance of invasion is unlikely to succeed in systems resembling our model specifications, because increasing the invader propagule size has little effect on invasion outcomes, except when the invader is almost as prevalent as the entirety of the resident community and even then disruption increases rather than augmentation, which is typically the desired outcome of such interventions. Each of the four invasion outcomes described here has a real-world equivalent in the human microbiota in which it is the desired state or outcome of an intervention. For example, resistance is typically the desired outcome of invasion into a healthy microbiota. There are many microbial communities that are known to resist invasion by pathogens, such as [1–5]. Our work suggests that interactions are likely a key component of this resistance, which would be favored in communities that inhibit the particular invader in question and have many facilitative interactions among residents. This implies that predicting, discovering, and improving upon successful microbiota interventions would be aided by a deeper, more species-specific understanding of the interactions that operate within microbial communities.

## Materials and methods

### Mathematical model

We use a mediator-explicit model that includes the species and the chemical environment that mediates interspecies interactions.

$$\frac{dC_{i}}{dt}=\sum_{j}[\beta_{ij}-\alpha_{ij}\frac{C_{i}}{C_{i}+K_{ij}}]S_{j}$$ (1A)

$$\frac{dS_{i}}{dt}=\left[r_{i0}+\sum_{j}r_{ij}\frac{C_{j}}{C_{j}+K_{ij}}\right]S_{i}$$ (1B)

In which *C*_*i*_ and *S*_*i*_ are the concentrations of the mediators and cell densities, respectively; *β*_*ij*_ and *α*_*ij*_ are production and consumption rates, respectively; and *K*_*ij*_ is the saturation concentration for uptake and growth rate influence. The following parameters are used throughout this work: *r*_*i*0_ has a uniform distribution between 0.08 and 0.12 hr^-1^; *r*_*ij*_’s amplitude has a uniform distribution between 0 and 0.2 hr^-1^, and its sign can be positive or negative with a probability that is specified in each case; *β*_*ij*_ has a uniform distribution between 0.05 and 0.15 fmole/cell per hour; *α*_*ij*_ has a uniform distribution between 0.25 and 0.75 fmole/cell per hour; and *K*_*ij*_ has a uniform distribution between 50 and 150 nM. The initial cell density is 10^4^ cells/ml, and the community is cyclically diluted back to this initial value when the density reaches 10^7^ cells/ml. This dilution scheme—replicating conventional growth situation in the lab—ensures that most shared resources are replenished during community growth [53]. Even though we use parameters similar to [53] throughout this manuscript, our conclusions are not sensitive to the detailed parameter values.

The model with explicit resource competition is different from the basic model in that it also includes a single resource that all species require for growth.

$$\frac{dC_{i}}{dt}=\sum_{j}[\beta_{ij}-\alpha_{ij}\frac{C_{i}}{C_{i}+K_{ij}}]S_{j}$$ (2A)

$$\frac{dS_{i}}{dt}=\left(\frac{R}{R+K_{R,i}}\right)\left(r_{i0}+\sum_{j}r_{ij}\frac{C_{i}}{C_{i}+K_{ij}}\right)S_{i}$$ (2B)

$$\frac{dR}{dt}=-\sum_{i}\alpha_{R,i}(\frac{R}{R+K_{R,i}})(r_{i0}+\sum_{j}r_{ij}\frac{C_{i}}{C_{i}+K_{ij}})S_{i}$$ (2C)

We have assumed that the consumption of resource *R* is directly proportional to the growth rate of species, with a given resource consumption rate *α*_*R*,*i*_. Resource *R* also affects the growth rate in a saturating fashion, with half-maximum concentration of *K*_*R*,*i*_. Unlike other mediators, the resource *R* is not produced by any of the species; it is supplied in the medium and thus replenished at dilution steps. For these simulations, dilution either happens when the density reaches 10^7^ cells/ml or after 80 hours. This choice is made to allow population turn-over even when the amount of resources in the environment is low to the level that the density does not reach the dilution threshold. The number of dilution cycles are adjusted in each case such that the culture experiences a total of 200 generations (regardless of the amount of resources available) to allow a fair comparison.

### Simulation platform

To find instances of resident communities, we simulated the enrichment process described in [53] in Matlab using the parameters listed in “Parameters used for simulations.”

For *in silico* invasion assays, we numerically solved the dynamic equations in Eqs 1 and 2 (without or with an explicit resource, respectively) using a forward Euler method implemented in Matlab. We chose the time-step for simulations to be at least ten times smaller than the shortest doubling time in each case. This choice offers a trade-off between accuracy and total simulation time. We have tested smaller time-steps and confirmed that the outcomes were not affected.

### Parameters used for simulations

Unless specified, the following parameters were used in simulations in this manuscript.

**Table:** 

**Parameter**	**Description**	**Value**
*N_c_*	Number of different species in the initial pool used for obtaining instances of resident communities	20
*N_m_*	Number of chemical mediators in the simulations that mediate the interactions among species	10
*N_s_*	Number of instances of simulations run for each case	1000
*N_gen_*	Number of generations simulated to obtain stable resident communities; also the number of generations simulated to assess invasion success	200
*F_inv_*	Range of invader introduction fractions examined for assessing invasion (20 points, equally spaced in log scale)	10^−4^ to 0.99
*N_inoc_ to N_dil_*	Range of total population density simulated (from inoculation to dilution threshold)	10^4^ to 10^7^ cells/ml
*N_ext_*	Extinction population density per species	0.1 cells/ml
*K_sat_*	Average chemical concentration threshold for saturation of chemical consumption and fitness influences (average of *K*_*ij*_ values)	10^4^ μM
*r_0_*	Basal net growth rate of community members, in the absence of interactions (uniform random distribution)	0.08–0.12 hr^-1^
*α_ij_*	Consumption rate of chemicals by species, per cell (uniform random distribution)	0.25–0.75 fmole.hr^-1^
*β_ij_*	Production rate of chemicals by species, per cell (uniform random distribution)	0.05–0.15 fmole.hr^-1^
*r_i0_*	Amplitude of the influence of chemicals on species’ growth rate (uniform random distribution)	0–0.2 hr^-1^
*f_fac_*	Fraction of non-neutral interactions among the initial pool of resident microbes that are facilitative	0.5
*r_i0,inv_*	Amplitude of the influence of chemicals on invader’s growth rate (uniform random distribution)	0–0.2 hr^-1^
*f_fac,inv_*	Fraction of non-neutral interactions affecting the invader that are facilitative	0.5
*q_p_*	The degree of connectivity of producer species to chemicals both for the resident members and the invader (forming a binomial network)	0.3
*q_c_*	The degree of connectivity of chemicals to species that they influence both for the resident members and the invader (forming a binomial network)	0.3
*R_0_*	Concentration of the limiting resource in fresh medium	10^6^ fmole/ml
*K_R_*	Average saturation concentration of the limiting resource	1 mM
*α_R_*	Consumption rate of limiting resource, per cell (uniform random distribution)	5–15 fmole.hr^-1^

### *In silico* invasion assay

To assess invasion success, we first assemble instances of stable resident communities *in silico*, following the enrichment procedure outlined in [53]. For each instance, we put together 20 species that are interacting through 10 chemical mediators. We simulate the dynamics in growth-dilution cycles for 200 generations to identify instances of stable *in silico* communities. We then introduce invaders at an initial fraction of 0.3%. We show that this fraction is a representative of the outcome at “small” propagule sizes (see Fig 2). We simulate the dynamics in growth-dilution cycles for an additional 200 generations. At the end of these simulations, we calculate the fraction of the invader cells in the resulting community. Based on the relative fraction of the invader between the initial and resulting communities across different propagule sizes, we categorize the invasion outcome as resistance, augmentation, displacement, or disruption. Resistance: resident community richness is maintained, invader goes extinct. Augmentation: resident community richness is maintained, invader population is maintained. Displacement: resident community richness drops, invader population is maintained. Disruption: resident community richness drops, invader goes extinct. To obtain reliable statistics about invasion outcomes, we repeat the process of assembling resident communities and challenging them with invaders for at least 1000 times.

## Supporting information

S1 Fig Only at large invader propagules colonization resistance is weakened. Here we have added confidence intervals to the graph, with all the parameters being similar to Fig 2. Confidence intervals for outcome frequencies are calculated using the Clopper-Pearson method (using binofit function in Matlab). 80% confidence intervals are plotted as a shaded region around each mean frequency. Number of instances examined: N_s_ = 1000.
(TIF)

S2 Fig Invaders with a higher basal growth rate shift invasion outcomes primarily from resistance to displacement. The pattern holds when the influence of mediators on the invader is (A) mostly inhibitory (*f*_*fac*,*inv*_ *=* 0.1), (B) equally facilitative or inhibitory (*f*_*fac*,*inv*_ *=* 0.5), or (C) mostly facilitative (*f*_*fac*,*inv*_ *=* 0.9). In all cases interactions among resident species are equally likely to be facilitative or inhibitory (*f*_*fac*_ = 0.5). Normalized basal growth rate of the invader is relative to resident species. Number of instances examined N_s_ = 1000.
(TIF)

S3 Fig Facilitation among resident species strengthens colonization resistance. Invasion success decreases when interactions among resident members are predominantly facilitation rather than inhibition. The interactions of resident species with the invader are (A) equally likely to be facilitative or inhibitory (*f*_*fac*,*inv*_ *=* 0.5) or (B) mostly facilitative (*f*_*fac*,*inv*_ *=* 0.9). In all cases interactions among resident species are equally likely to be facilitative or inhibitory (*f*_*fac*_ = 0.5). Normalized basal growth rate of the invader is 1.5 (compared to resident members). Number of instances examined N_s_ = 1000.
(TIF)

S4 Fig Invader connectivity affects invasion outcomes. (A) When the chance of mediators influencing the invader increases, colonization resistance is strengthened, as expected. (B) When the chance of the invader producing chemical mediators increases, augmentation becomes more likely. Interactions between resident species and the invader are mostly inhibitory (*f*_*fac*,*inv*_ *=* 0.1). Interactions among resident species are equally likely to be facilitative or inhibitory (*f*_*fac*_ = 0.5). Normalized basal growth rate of the invader is 1.5 (compared to resident members). Number of instances examined N_s_ = 1000.
(TIF)

S5 Fig The effective interaction between the resident community and the invader alters the outcome of invasion. We expanded the results in Fig 5 (all parameters kept the same) to demonstrate all eight possible combinations of keeping or removing production, consumption, or mediator influence. Interactions among resident species are equally likely to be facilitative or inhibitory (*f*_*fac*_ *=* 0.5). The influence of residents on the invader is mostly inhibitory (*f*_*fac*,*inv*_ *=* 0.1). Invader has a normalized basal growth rate of 1.5. Number of instances examined N_s_ = 10000.
(TIF)

S6 Fig Colonization resistance is weakened when the mediators that inhibit the invader are depleted. (A) When the production of mediators increases, colonization resistance is strengthened. (B) When the consumption of the mediators is increased, mediators are depleted and thus colonization resistance is weakened. Interactions between resident species and the invader are mostly inhibitory (*f*_*fac*,*inv*_ *=* 0.1). Interactions among resident species are equally likely to be facilitative or inhibitory (*f*_*fac*_ = 0.5). Normalized basal growth rate of the invader is 1.5 (compared to resident members). Number of instances examined N_s_ = 1000.
(TIF)

S7 Fig With explicit resource competition, the extent of resource limitation only marginally influences the outcomes. Interactions between resident species and the invader are mostly inhibitory (*f*_*fac*,*inv*_ *=* 0.1). Interactions among resident species are equally likely to be facilitative or inhibitory (*f*_*fac*_ = 0.5). Normalized basal growth rate of the invader is 1.5 (compared to resident members). The invader is introduced at 0.3% of the resident community size. The amount of the limiting resource is varied between 10^5^ and 10^7^ fmole/ml. Each invasion assay is run for as many dilution rounds as needed to reach 200 generations of total community growth. Number of instances examined N_s_ = 1000.
(TIF)

S8 Fig Only invader propagules comparable to community size can weaken colonization resistance, when explicit resource competition in included. As the normalized propagule size increases, the probability of resistance decreases, the probability of disruption increases, and the probability of augmentation or displacement remains approximately constant. Number of instances examined N_s_ = 1000. Interactions among resident members are equally likely to be facilitative or inhibitory (*f*_*fac*_ *=* 0.5). Interactions between resident members and the invader are mostly inhibitory (*f*_*fac*,*inv*_ = 0.1). *r*_*0*,*inv*_/*r*_*0*,*res*_ = 1.5. The amount of the limiting resource is set at 10^6^ fmole/ml.
(TIF)

S9 Fig Invaders with a higher basal growth rate shift invasion outcomes primarily from resistance to displacement, when a single limiting resource is added to the model. The influence of mediators on the invader is mostly inhibitory (*f*_*fac*,*inv*_ *=* 0.1). Interactions among resident species are equally likely to be facilitative or inhibitory (*f*_*fac*_ = 0.5). Normalized basal growth rate of the invader is relative to resident species. Number of instances examined N_s_ = 1000. The amount of the limiting resource is set at 10^6^ fmole/ml.
(TIF)

S10 Fig When resident species facilitate the invader, colonization resistance is weakened. Invasion success drastically increases when we switch the interactions that influence the invader from inhibition to facilitation. Number of instances examined N_s_ = 1000. Interactions among resident members are equally likely to be facilitative or inhibitory (*f*_*fac*_ *=* 0.5). Normalized basal growth rate of the invader is 1.5. Normalized introduced propagule size is 0.3%. The amount of the limiting resource is set at 10^6^ fmole/ml.
(TIF)

S11 Fig Facilitation among resident species strengthens colonization resistance, even with explicit resource competition. Invasion success decreases when interactions among resident species are predominantly facilitation rather than inhibition. The interactions between resident species and the invader are mostly inhibitory (*f*_*fac*,*inv*_ *=* 0.1). Normalized basal growth rate of the invader is 1.5 (compared to resident members). Normalized introduced propagule size is 0.3%. The amount of the limiting resource is set at 10^6^ fmole/ml.
(TIF)

## References

[1] Fehervari Z. Mechanisms of colonization resistance. Nat Res 2019 2019.

[2] Brugger SD, Bomar L, Lemon KP. Commensal–Pathogen Interactions along the Human Nasal Passages. PLOS Pathog. 2016;12: e1005633 10.1371/journal.ppat.1005633 27389401 PMC4936728

[3] Man WH, de Steenhuijsen Piters WAA, Bogaert D. The microbiota of the respiratory tract: gatekeeper to respiratory health. Nat Rev Microbiol. 2017;15: 259–270. 10.1038/nrmicro.2017.14 28316330 PMC7097736

[4] Buffie CG, Pamer EG. Microbiota-mediated colonization resistance against intestinal pathogens. Nat Rev Immunol. 2013;13: 790–801. 10.1038/nri3535 24096337 PMC4194195

[5] Stein RR, Bucci V, Toussaint NC, Buffie CG, Rätsch G, Pamer EG, et al Ecological Modeling from Time-Series Inference: Insight into Dynamics and Stability of Intestinal Microbiota. PLoS Comput Biol. 2013;9: e1003388 10.1371/journal.pcbi.1003388 24348232 PMC3861043

[6] He X, McLean JS, Guo L, Lux R, Shi W. The social structure of microbial community involved in colonization resistance. ISME J. 2014;8: 564–574. 10.1038/ismej.2013.172 24088624 PMC3930314

[7] Bomar L, Brugger SD, Yost BH, Davies SS, Lemon KP. Corynebacterium accolens Releases Antipneumococcal Free Fatty Acids from Human Nostril and Skin Surface Triacylglycerols. mBio. 2016;7: e01725–15. 10.1128/mBio.01725-15 26733066 PMC4725001

[8] Uehara Y, Nakama H, Agematsu K, Uchida M, Kawakami Y, Abdul Fattah A, et al Bacterial interference among nasal inhabitants: eradication of Staphylococcus aureus from nasal cavities by artificial implantation of Corynebacterium sp. J Hosp Infect. 2000;44: 127–133. 10.1053/jhin.1999.0680 10662563

[9] Iwase T, Uehara Y, Shinji H, Tajima A, Seo H, Takada K, et al Staphylococcus epidermidis Esp inhibits Staphylococcus aureus biofilm formation and nasal colonization. Nature. 2010;465: 346–349. 10.1038/nature09074 20485435

[10] Taur Y, Pamer EG. Harnessing Microbiota to Kill a Pathogen: Fixing the microbiota to treat Clostridium difficile infections. Nat Med. 2014;20: 246–247. 10.1038/nm.3492 24603796 PMC4542075

[11] Vaughn BP, Vatanen T, Allegretti JR, Bai A, Xavier RJ, Korzenik J, et al Increased Intestinal Microbial Diversity Following Fecal Microbiota Transplant for Active Crohn’s Disease. Inflamm Bowel Dis. 2016;22: 2182–2190. 10.1097/MIB.0000000000000893 27542133 PMC4995064

[12] Costello SP, Hughes PA, Waters O, Bryant RV, Vincent AD, Blatchford P, et al Effect of Fecal Microbiota Transplantation on 8-Week Remission in Patients With Ulcerative Colitis: A Randomized Clinical Trial. JAMA. 2019;321: 156–164. 10.1001/jama.2018.20046 30644982 PMC6439766

[13] Sherman MP, Zaghouani H, Niklas V. Gut microbiota, the immune system, and diet influence the neonatal gut–brain axis. Pediatr Res. 2015;77: 127–135. 10.1038/pr.2014.161 25303278

[14] Gritz EC, Bhandari V. The Human Neonatal Gut Microbiome: A Brief Review. Front Pediatr. 2015;3 10.3389/fped.2015.00017 25798435 PMC4350424

[15] Sohn K, Underwood MA. Prenatal and postnatal administration of prebiotics and probiotics. Semin Fetal Neonatal Med. 2017;22: 284–289. 10.1016/j.siny.2017.07.002 28720399 PMC5618799

[16] Mastromarino P, Vitali B, Mosca L. Bacterial vaginosis: a review on clinical trials with probiotics.: 10. 23912864

[17] Gareau MG, Sherman PM, Walker WA. Probiotics and the gut microbiota in intestinal health and disease. Nat Rev Gastroenterol Hepatol. 2010;7: 503–514. 10.1038/nrgastro.2010.117 20664519 PMC4748966

[18] Buffie CG, Bucci V, Stein RR, McKenney PT, Ling L, Gobourne A, et al Precision microbiome reconstitution restores bile acid mediated resistance to Clostridium difficile. Nature. 2014 10.1038/nature13828 25337874 PMC4354891

[19] Kearney SM, Gibbons SM, Erdman SE, Alm EJ. Orthogonal Dietary Niche Enables Reversible Engraftment of a Gut Bacterial Commensal. Cell Rep. 2018;24: 1842–1851. 10.1016/j.celrep.2018.07.032 30110640 PMC6724203

[20] Jousset A, Schulz W, Scheu S, Eisenhauer N. Intraspecific genotypic richness and relatedness predict the invasibility of microbial communities. ISME J. 2011;5: 1108–1114. 10.1038/ismej.2011.9 21346790 PMC3146292

[21] De Roy K, Marzorati M, Negroni A, Thas O, Balloi A, Fava F, et al Environmental conditions and community evenness determine the outcome of biological invasion. Nat Commun. 2013;4 10.1038/ncomms2392 23340423

[22] Maltby R, Leatham-Jensen MP, Gibson T, Cohen PS, Conway T. Nutritional Basis for Colonization Resistance by Human Commensal Escherichia coli Strains HS and Nissle 1917 against E. coli O157:H7 in the Mouse Intestine. Ibekwe AM, editor. PLoS ONE. 2013;8: e53957 10.1371/journal.pone.0053957 23349773 PMC3547972

[23] Li S peng, Tan J, Yang X, Ma C, Jiang L. Niche and fitness differences determine invasion success and impact in laboratory bacterial communities. ISME J. 2019;13: 402–412. 10.1038/s41396-018-0283-x 30254322 PMC6331569

[24] Hooper DU, Dukes JS. Functional composition controls invasion success in a California serpentine grassland. J Ecol. 2010;98: 764–777. 10.1111/j.1365-2745.2010.01673.x

[25] Papacostas KJ, Rielly-Carroll EW, Georgian SE, Long DJ, Princiotta SD, Quattrini AM, et al Biological mechanisms of marine invasions. Mar Ecol Prog Ser. 2017;565: 251–268. 10.3354/meps12001

[26] Mallon CA, Van Elsas JD, Salles JF. Microbial invasions: The process, patterns, and mechanisms Trends in Microbiology. Elsevier Ltd; 2015 pp. 719–729. 10.1016/j.tim.2015.07.013 26439296

[27] Litvak Y, Bäumler AJ. The founder hypothesis: A basis for microbiota resistance, diversity in taxa carriage, and colonization resistance against pathogens. PLOS Pathog. 2019;15: e1007563 10.1371/journal.ppat.1007563 30789972 PMC6383860

[28] Vila JCC, Jones ML, Patel M, Bell T, Rosindell J. Uncovering the rules of microbial community invasions. Nat Ecol Evol. 2019;3: 1162–1171. 10.1038/s41559-019-0952-9 31358951

[29] Shea K, Chesson P. Community ecology theory as a framework for biological invasions. Trends Ecol Evol. 2002;17: 170–176. 10.1016/S0169-5347(02)02495-3

[30] Pamer EG. Resurrecting the intestinal microbiota to combat antibiotic-resistant pathogens. Science. 2016;352: 535–8. 10.1126/science.aad9382 27126035 PMC4984266

[31] Lawley TD, Walker AW. Intestinal colonization resistance. Immunology. 2013;138: 1–11. 10.1111/j.1365-2567.2012.03616.x 23240815 PMC3533696

[32] Elton CS. No Title. The Ecology of Invasions by Animals and Plants. London, England: Methuen; 1958 pp. 145–153.

[33] Lockwood JL, Hoopes MF, Marchetti MP. Invasion ecology. 2nd ed Wiley-Blackwell; 2013.

[34] Carlton JT. Pattern, process, and prediction in marine invasion ecology. Biol Conserv. 1996;78: 97–106. 10.1016/0006-3207(96)00020-1

[35] Catford JA, Jansson R, Nilsson C. Reducing redundancy in invasion ecology by integrating hypotheses into a single theoretical framework. Divers Distrib. 2009;15: 22–40. 10.1111/j.1472-4642.2008.00521.x

[36] van der Putten WH, Klironomos JN, Wardle DA. Microbial ecology of biological invasions. ISME J. 2007;1: 28–37. 10.1038/ismej.2007.9 18043611

[37] Altieri AH, van Wesenbeeck BK, Bertness MD, Silliman BR. Facilitation cascade drives positive relationship between native biodiversity and invasion success. Ecology. 2010;91: 1269–1275. 10.1890/09-1301.1 20503860

[38] Stachowicz J, Byrnes J. Species diversity, invasion success, and ecosystem functioning: disentangling the influence of resource competition, facilitation, and extrinsic factors. Mar Ecol Prog Ser. 2006;311: 251–262. 10.3354/meps311251

[39] Traveset A, Richardson DM. Mutualistic Interactions and Biological Invasions. Annu Rev Ecol Evol Syst. 2014;45: 89–113. 10.1146/annurev-ecolsys-120213-091857

[40] Adams MJ, Pearl CA, Bruce Bury R. Indirect facilitation of an anuran invasion by non-native fishes. Ecol Lett. 2003;6: 343–351. 10.1046/j.1461-0248.2003.00435.x

[41] Rakoff-Nahoum S, Coyne MJ, Comstock LE. An ecological network of polysaccharide utilization among human intestinal symbionts. Curr Biol CB. 2014;24: 40–49. 10.1016/j.cub.2013.10.077 24332541 PMC3924574

[42] Czárán TL, Hoekstra RF, Pagie L. Chemical warfare between microbes promotes biodiversity. Proc Natl Acad Sci U S A. 2002;99: 786–790. 10.1073/pnas.012399899 11792831 PMC117383

[43] Ponomarova O, Patil KR. Metabolic interactions in microbial communities: untangling the Gordian knot. Curr Opin Microbiol. 2015;27: 37–44. 10.1016/j.mib.2015.06.014 26207681

[44] Kettle H, Louis P, Holtrop G, Duncan SH, Flint HJ. Modelling the emergent dynamics and major metabolites of the human colonic microbiota. Environ Microbiol. 2015;17: 1615–1630. 10.1111/1462-2920.12599 25142831

[45] D’Acunto B, Esposito G, Frunzo L, Luongo V, Mattei MR, Pirozzi F. Microbial colonization of anaerobic biofilms: a mathematical model. 2017; 10.

[46] D’Acunto B, Frunzo L, Klapper I, Mattei MR. Modeling multispecies biofilms including new bacterial species invasion. Math Biosci. 2015;259: 20–26. 10.1016/j.mbs.2014.10.009 25447810

[47] Delboni RR, Yang HM. Mathematical Model of Interaction Between Bacteriocin-Producing Lactic Acid Bacteria and Listeria. Part 2: Bifurcations and Applications. Bull Math Biol. 2017;79: 2273–2301. 10.1007/s11538-017-0330-1 28799082

[48] Delboni RR, Yang HM. Mathematical Model of Interaction Between Bacteriocin-Producing Lactic Acid Bacteria and Listeria. Part 1: Steady States and Thresholds. Bull Math Biol. 2017;79: 1637–1661. 10.1007/s11538-017-0302-5 28597170

[49] Murray JD. Invasion by Invitation: Rhizobial Infection in Legumes. Mol Plant Microbe Interact. 2011;24: 631–639. 10.1094/MPMI-08-10-0181 21542766

[50] La Pierre KJ, Simms EL, Tariq M, Zafar M, Porter SS. Invasive legumes can associate with many mutualists of native legumes, but usually do not. Ecol Evol. 2017;7: 8599–8611. 10.1002/ece3.3310 29075475 PMC5648655

[51] Boureau H, Hartmann L, Karjalainen T, Rowland I, Wilkinson MHF. Models to Study Colonisation and Colonisation Resistance. Microb Ecol Health Dis. 2000;12: 247–258. 10.1080/08910600050216246

[52] Momeni B, Xie L, Shou W. Lotka-Volterra pairwise modeling fails to capture diverse pairwise microbial interactions. eLife. 2017;6: e25051 10.7554/eLife.25051 28350295 PMC5469619

[53] Niehaus L, Boland I, Liu M, Chen K, Fu D, Henckel C, et al Microbial coexistence through chemical-mediated interactions. Nat Commun. 2019;10: 2052 10.1038/s41467-019-10062-x 31053707 PMC6499789

[54] Kinnunen M, Dechesne A, Proctor C, Hammes F, Johnson D, Quintela-Baluja M, et al A conceptual framework for invasion in microbial communities. ISME J. 2016;10: 2773–2775. 10.1038/ismej.2016.75 27137125 PMC5148196

[55] Momeni B, Brileya KA, Fields MW, Shou W. Strong inter-population cooperation leads to partner intermixing in microbial communities. eLife. 2013;2: e00230 10.7554/eLife.00230 23359860 PMC3552619

[56] Gordon DM, Riley MA. A theoretical and empirical investigation of the invasion dynamics of colicinogeny. Microbiology,. 1999;145: 655–661. 10.1099/13500872-145-3-655 10217499

[57] Brown SP, Le Chat L, De Paepe M, Taddei F. Ecology of Microbial Invasions: Amplification Allows Virus Carriers to Invade More Rapidly When Rare. Curr Biol. 2006;16: 2048–2052. 10.1016/j.cub.2006.08.089 17055985

[58] Acosta F, Zamor RM, Najar FZ, Roe BA, Hambright KD. Dynamics of an experimental microbial invasion. Proc Natl Acad Sci. 2015;112: 11594–11599. 10.1073/pnas.1505204112 26324928 PMC4577205

[59] Jones ML, Ramoneda J, Rivett DW, Bell T. Biotic resistance shapes the influence of propagule pressure on invasion success in bacterial communities. Ecology. 2017;98: 1743–1749. 10.1002/ecy.1852 28397255

[60] Mehnert G, Leunert F, Cirés S, Jöhnk KD, Rücker J, Nixdorf B, et al Competitiveness of invasive and native cyanobacteria from temperate freshwaters under various light and temperature conditions. J Plankton Res. 2010;32: 1009–1021. 10.1093/plankt/fbq033

[61] Spaulding SA, Elwell E. Increase in nuisance blooms and geographic expansion of the freshwater diatom Didymosphenia geminata. Increase Nuis Blooms Geogr Expans Freshw Diatom Didymosphenia Geminata. Reston, VA: U.S. Geological Survey; 2007 Report No.: 2007–1425. 10.3133/ofr20071425

[62] Isvánovics V, Shafik HM, Présing M, Juhos S. Growth and phosphate uptake kinetics of the cyanobacterium, Cylindrospermopsis raciborskii (Cyanophyceae) in throughflow cultures. Freshw Biol. 2000;43: 257–275. 10.1046/j.1365-2427.2000.00549.x

[63] Li M, Wei Z, Wang J, Jousset A, Friman V-P, Xu Y, et al Facilitation promotes invasions in plant-associated microbial communities. Ecol Lett. 2019;22: 149–158. 10.1111/ele.13177 30460736

[64] Fridley JD, Stachowicz JJ, Naeem S, Sax DF, Seabloom EW, Smith MD, et al The Invasion Paradox: Reconciling Pattern and Process in Species Invasions. Ecology. 2007;88: 3–17. 10.1890/0012-9658(2007)88[3:tiprpa]2.0.co;2 17489447

[65] Xing J, Jia X, Wang H, Ma B, Salles JF, Xu J. The legacy of bacterial invasions on soil native communities. Environ Microbiol. n/a. 10.1111/1462-2920.15086 32419297

[66] Ruhe ZC, Low DA, Hayes CS. Bacterial contact-dependent growth inhibition. Trends Microbiol. 2013;21: 230–237. 10.1016/j.tim.2013.02.003 23473845 PMC3648609

[67] Zoued A, Brunet YR, Durand E, Aschtgen M-S, Logger L, Douzi B, et al Architecture and assembly of the Type VI secretion system. Biochim Biophys Acta BBA—Mol Cell Res. 2014;1843: 1664–1673. 10.1016/j.bbamcr.2014.03.018 24681160

[68] Littman DR, Pamer EG. Role of the Commensal Microbiota in Normal and Pathogenic Host Immune Responses. Cell Host Microbe. 2011;10: 311–323. 10.1016/j.chom.2011.10.004 22018232 PMC3202012

