# Supplementary figures and images for: The impact of interactions on invasion and colonization resistance in microbial communities
Source: PLoS Comput Biol. 2021 Jan 22;17(1):e1008643. doi: 10.1371/journal.pcbi.1008643 (PMC7857599; doi:10.1371/journal.pcbi.1008643)

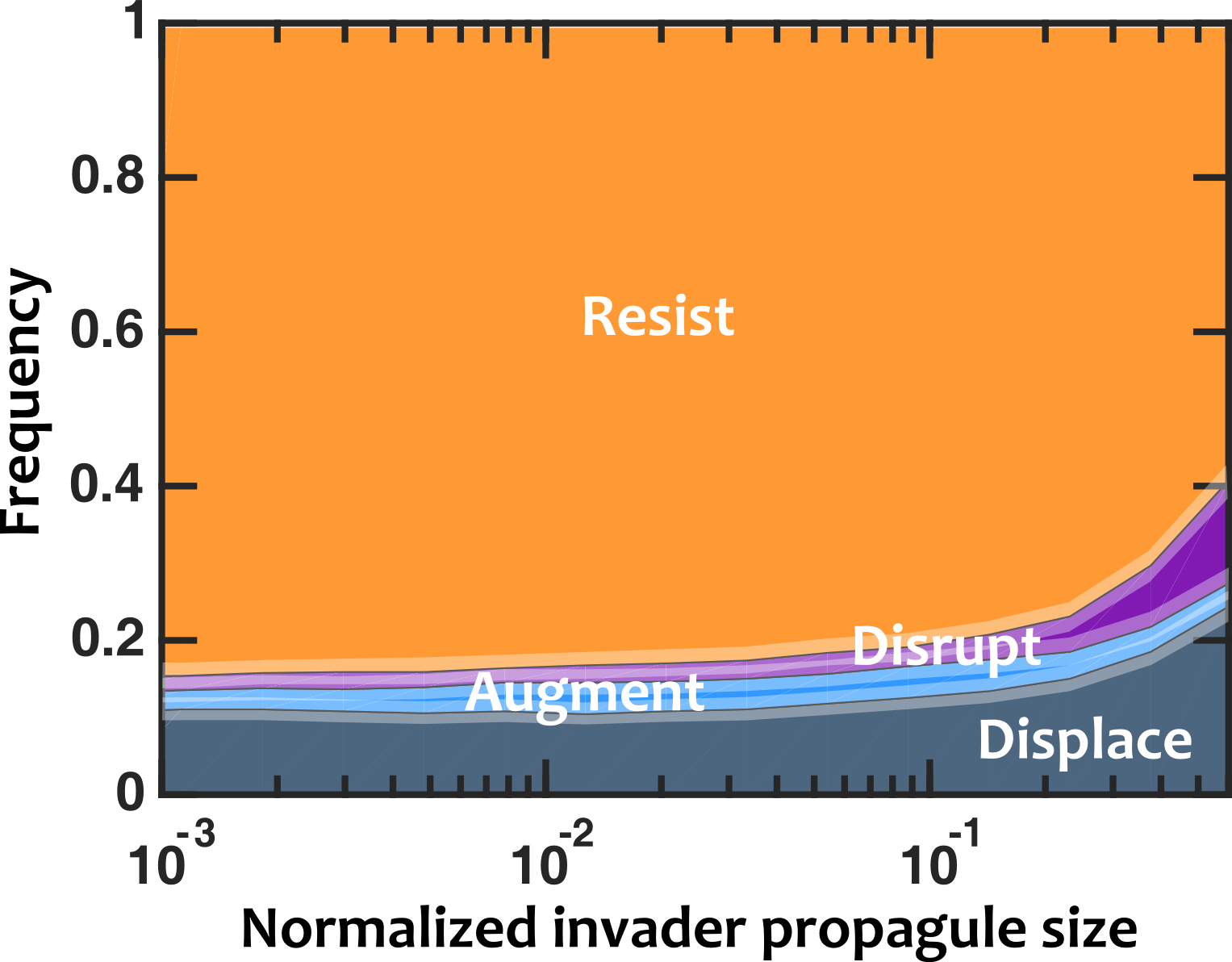

Supplement: S1 Fig — Here we have added confidence intervals to the graph, with all the parameters being similar to Fig 2. Confidence intervals for outcome frequencies are calculated using the Clopper-Pearson method (using binofit function in Matlab). 80% confidence intervals are plotted as a shaded region around each mean frequency. Number of instances examined: Ns = 1000. (TIF) [file pcbi.1008643.s001.tif]

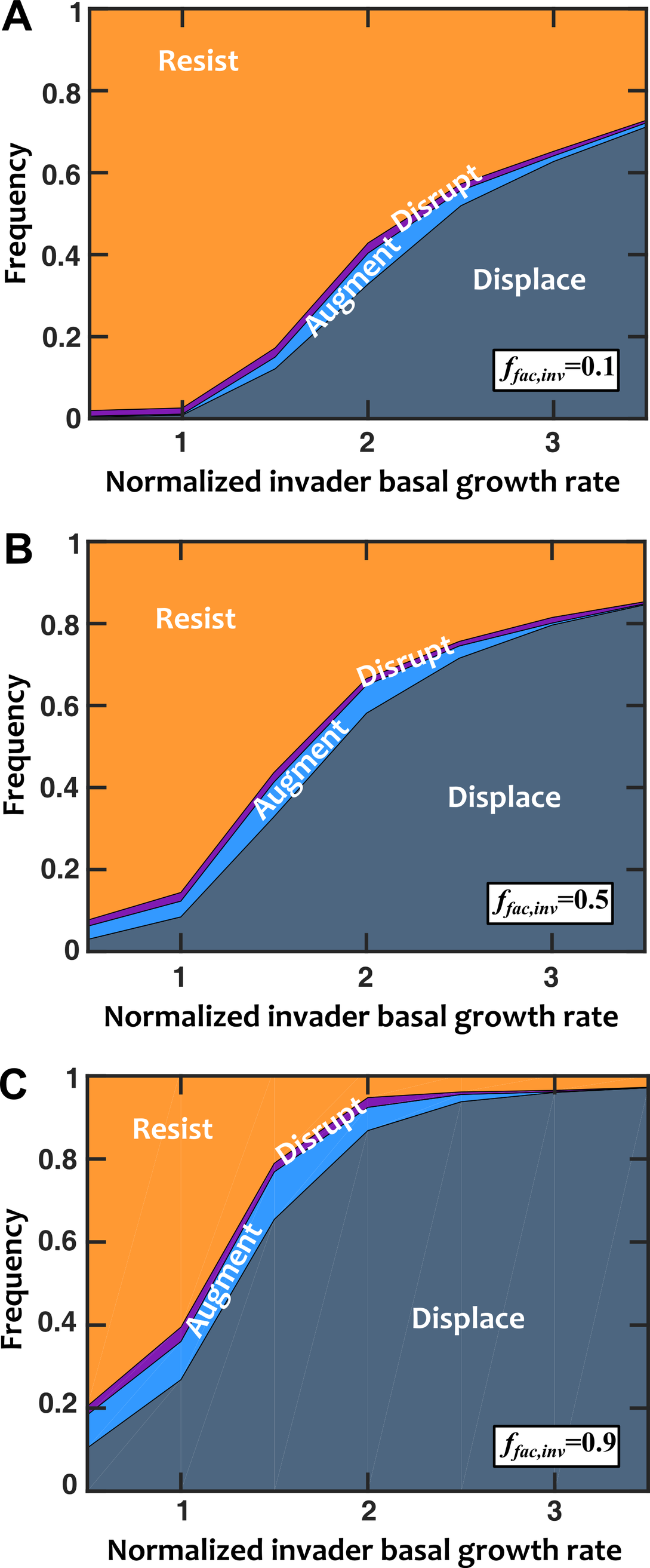

Supplement: S2 Fig — The pattern holds when the influence of mediators on the invader is (A) mostly inhibitory (ffac,inv = 0.1), (B) equally facilitative or inhibitory (ffac,inv = 0.5), or (C) mostly facilitative (ffac,inv = 0.9). In all cases interactions among resident species are equally likely to be facilitative or inhibitory (ffac = 0.5). Normalized basal growth rate of the invader is relative to resident species. Number of instances examined Ns = 1000. (TIF) [file pcbi.1008643.s002.tif]

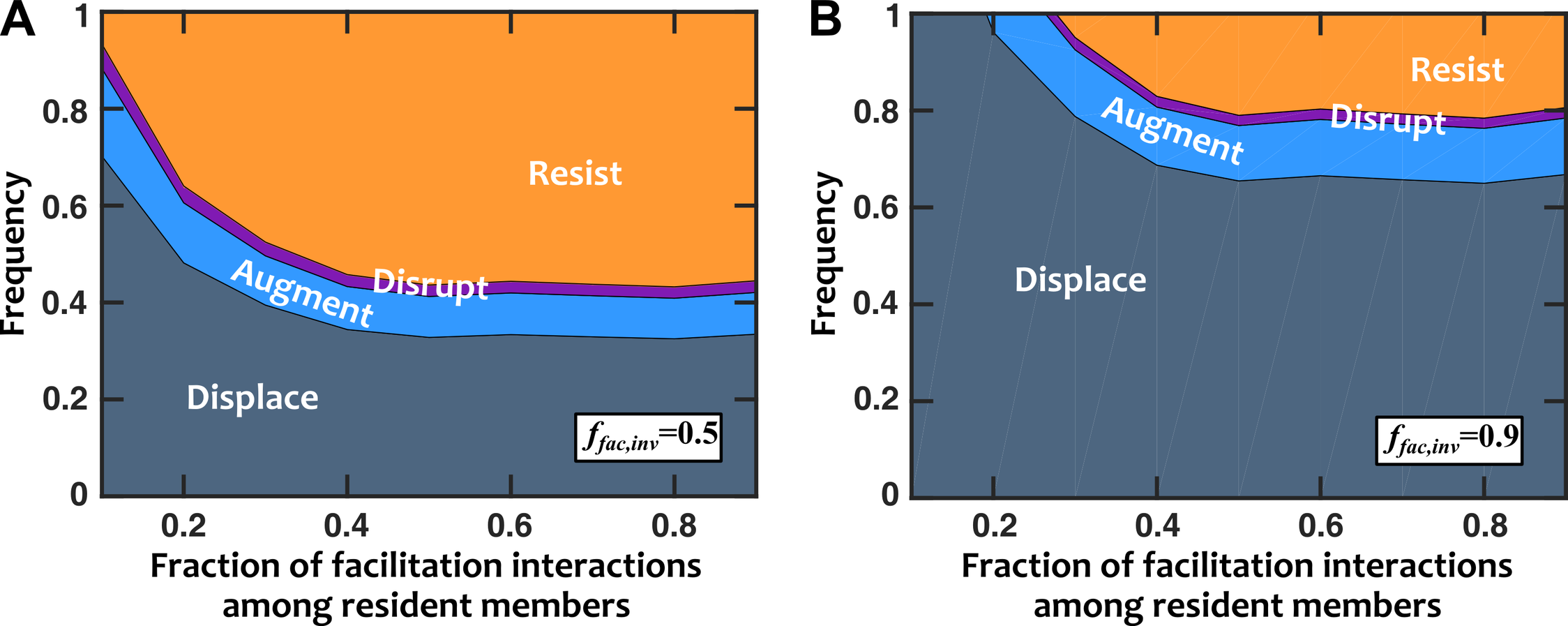

Supplement: S3 Fig — Invasion success decreases when interactions among resident members are predominantly facilitation rather than inhibition. The interactions of resident species with the invader are (A) equally likely to be facilitative or inhibitory (ffac,inv = 0.5) or (B) mostly facilitative (ffac,inv = 0.9). In all cases interactions among resident species are equally likely to be facilitative or inhibitory (ffac = 0.5). Normalized basal growth rate of the invader is 1.5 (compared to resident members). Number of instances examined Ns = 1000. (TIF) [file pcbi.1008643.s003.tif]

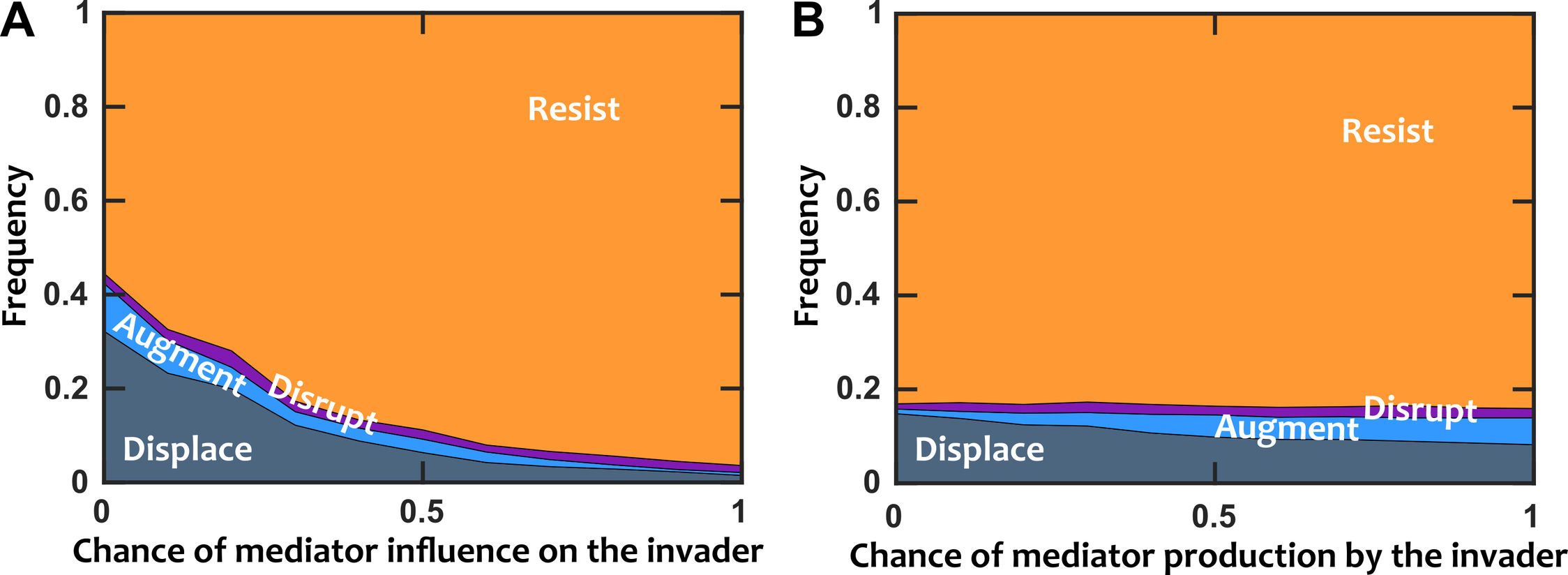

Supplement: S4 Fig — (A) When the chance of mediators influencing the invader increases, colonization resistance is strengthened, as expected. (B) When the chance of the invader producing chemical mediators increases, augmentation becomes more likely. Interactions between resident species and the invader are mostly inhibitory (ffac,inv = 0.1). Interactions among resident species are equally likely to be facilitative or inhibitory (ffac = 0.5). Normalized basal growth rate of the invader is 1.5 (compared to resident members). Number of instances examined Ns = 1000. (TIF) [file pcbi.1008643.s004.tif]

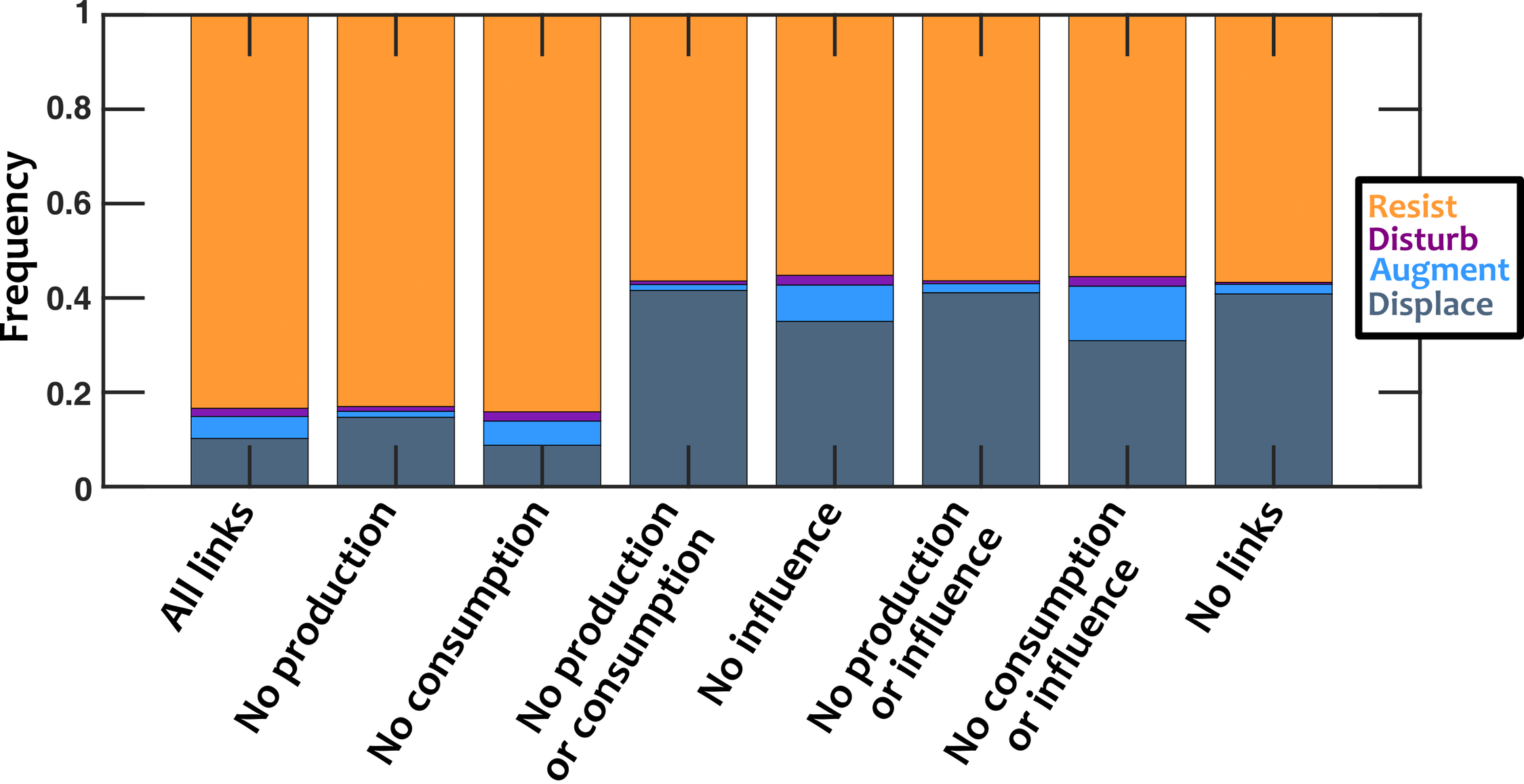

Supplement: S5 Fig — We expanded the results in Fig 5 (all parameters kept the same) to demonstrate all eight possible combinations of keeping or removing production, consumption, or mediator influence. Interactions among resident species are equally likely to be facilitative or inhibitory (ffac = 0.5). The influence of residents on the invader is mostly inhibitory (ffac,inv = 0.1). Invader has a normalized basal growth rate of 1.5. Number of instances examined Ns = 10000. (TIF) [file pcbi.1008643.s005.tif]

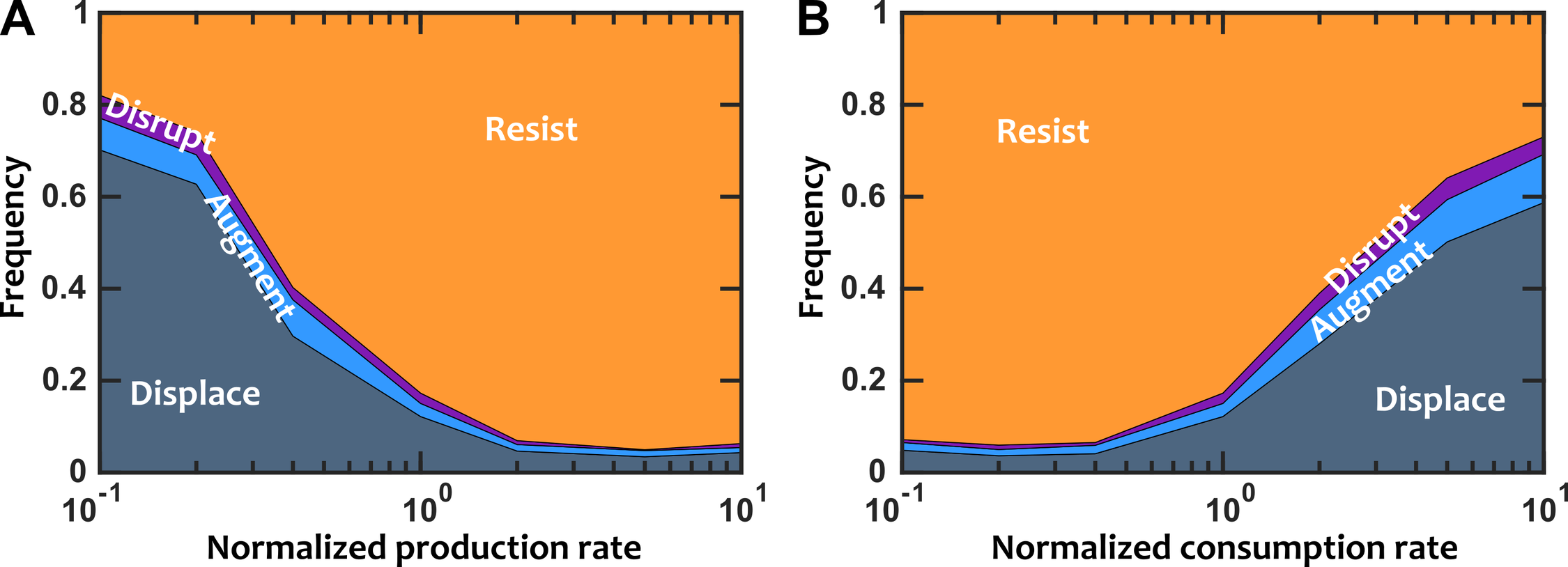

Supplement: S6 Fig — (A) When the production of mediators increases, colonization resistance is strengthened. (B) When the consumption of the mediators is increased, mediators are depleted and thus colonization resistance is weakened. Interactions between resident species and the invader are mostly inhibitory (ffac,inv = 0.1). Interactions among resident species are equally likely to be facilitative or inhibitory (ffac = 0.5). Normalized basal growth rate of the invader is 1.5 (compared to resident members). Number of instances examined Ns = 1000. (TIF) [file pcbi.1008643.s006.tif]

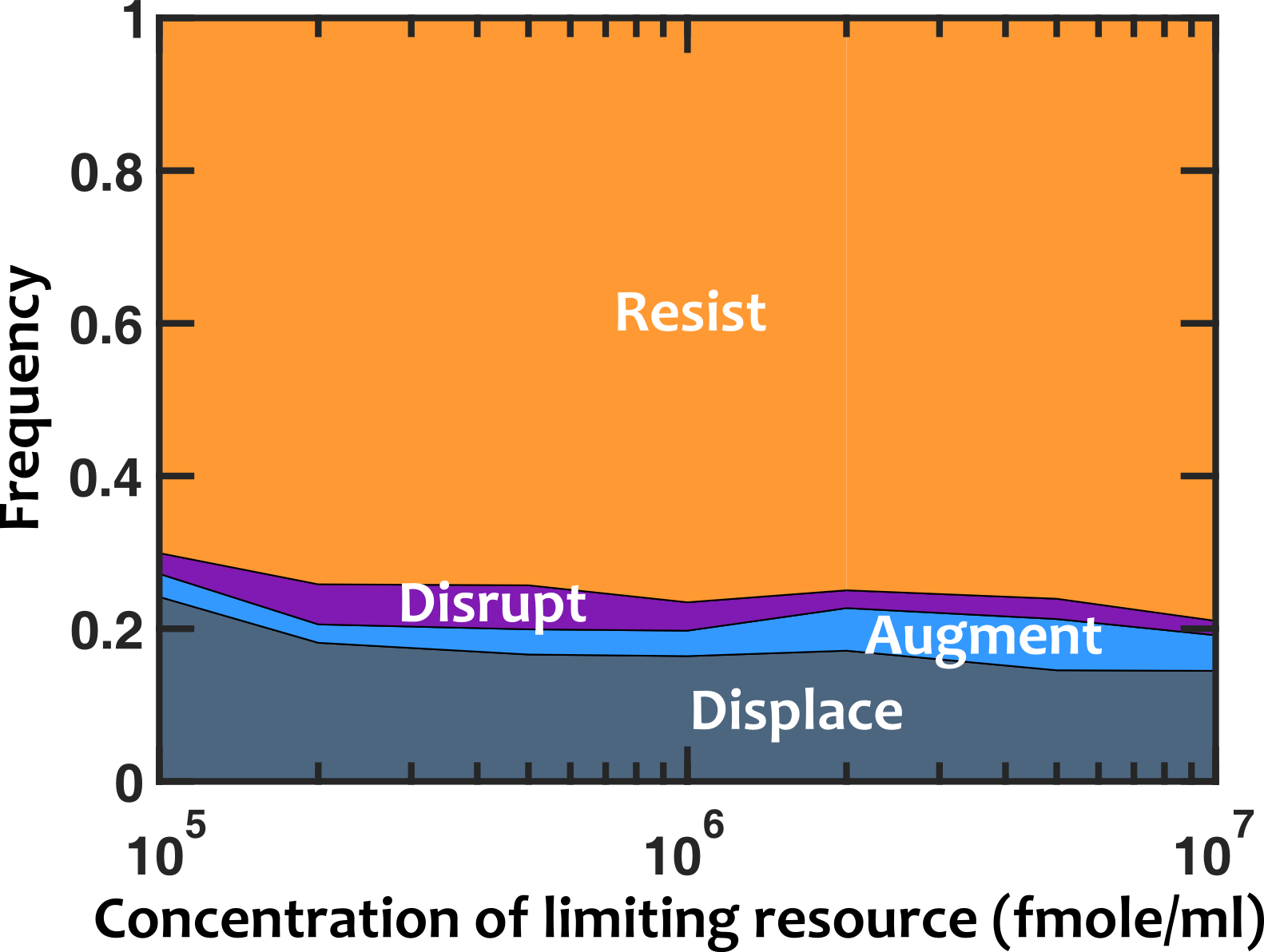

Supplement: S7 Fig — Interactions between resident species and the invader are mostly inhibitory (ffac,inv = 0.1). Interactions among resident species are equally likely to be facilitative or inhibitory (ffac = 0.5). Normalized basal growth rate of the invader is 1.5 (compared to resident members). The invader is introduced at 0.3% of the resident community size. The amount of the limiting resource is varied between 105 and 107 fmole/ml. Each invasion assay is run for as many dilution rounds as needed to reach 200 generations of total community growth. Number of instances examined Ns = 1000. (TIF) [file pcbi.1008643.s007.tif]

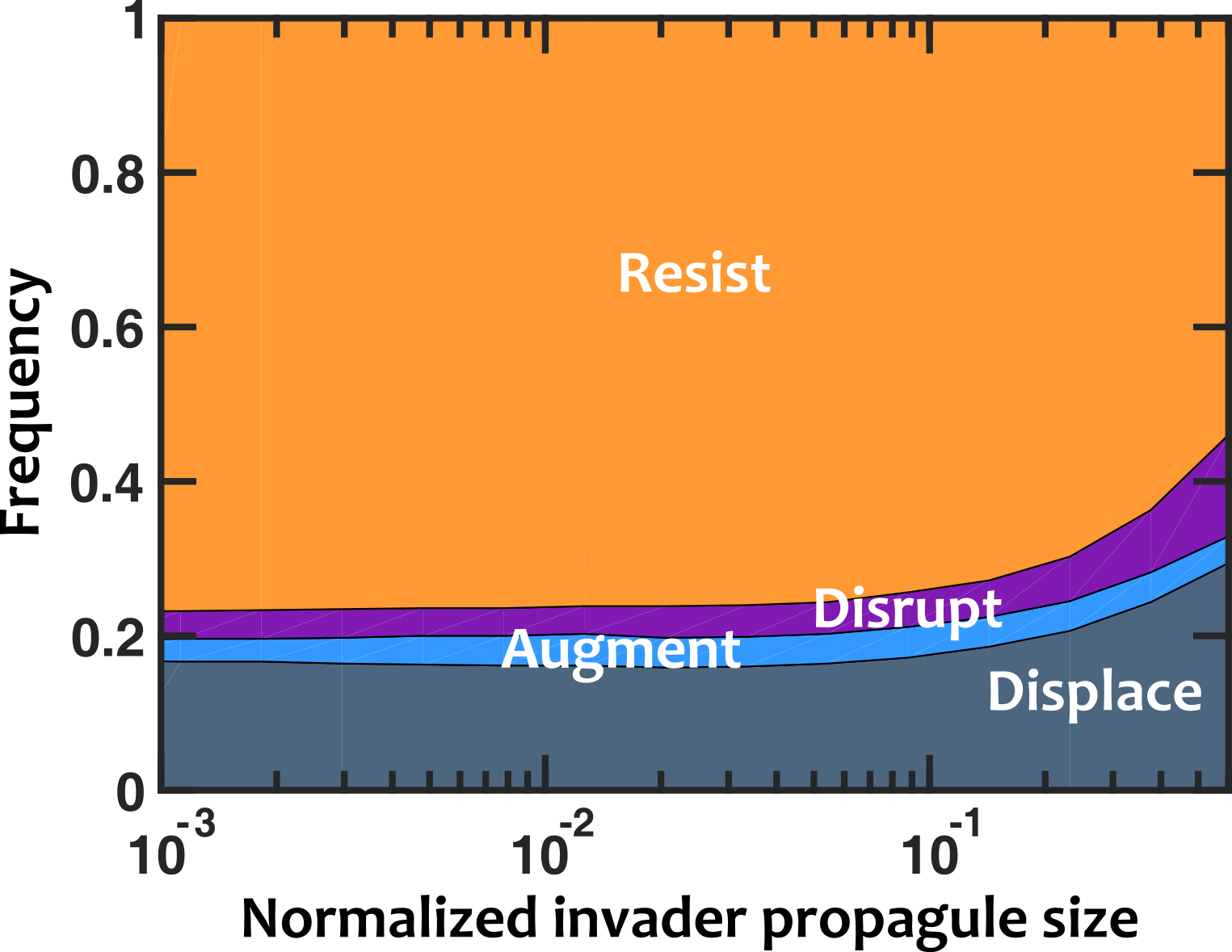

Supplement: S8 Fig — As the normalized propagule size increases, the probability of resistance decreases, the probability of disruption increases, and the probability of augmentation or displacement remains approximately constant. Number of instances examined Ns = 1000. Interactions among resident members are equally likely to be facilitative or inhibitory (ffac = 0.5). Interactions between resident members and the invader are mostly inhibitory (ffac,inv = 0.1). r0,inv/r0,res = 1.5. The amount of the limiting resource is set at 106 fmole/ml. (TIF) [file pcbi.1008643.s008.tif]

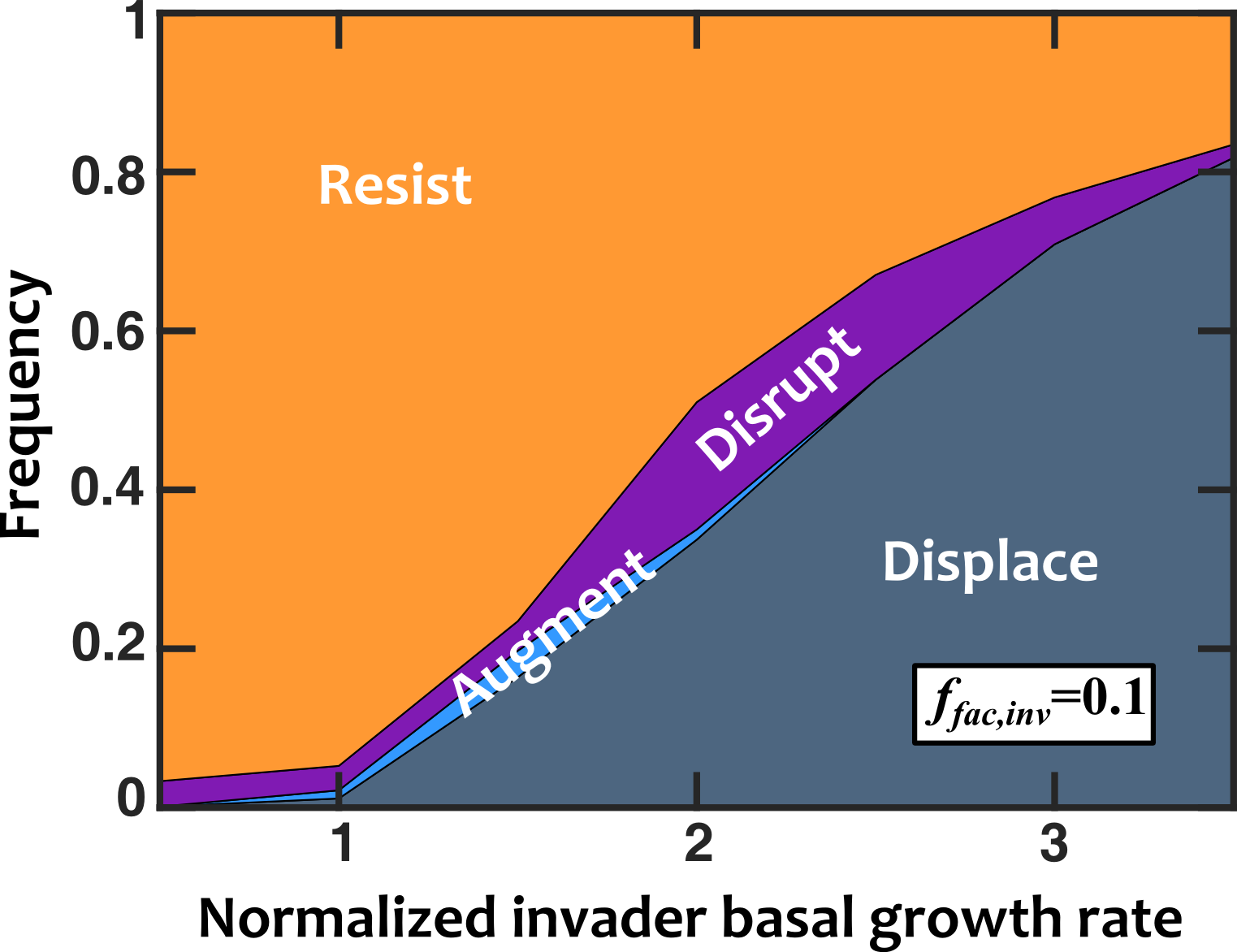

Supplement: S9 Fig — The influence of mediators on the invader is mostly inhibitory (ffac,inv = 0.1). Interactions among resident species are equally likely to be facilitative or inhibitory (ffac = 0.5). Normalized basal growth rate of the invader is relative to resident species. Number of instances examined Ns = 1000. The amount of the limiting resource is set at 106 fmole/ml. (TIF) [file pcbi.1008643.s009.tif]

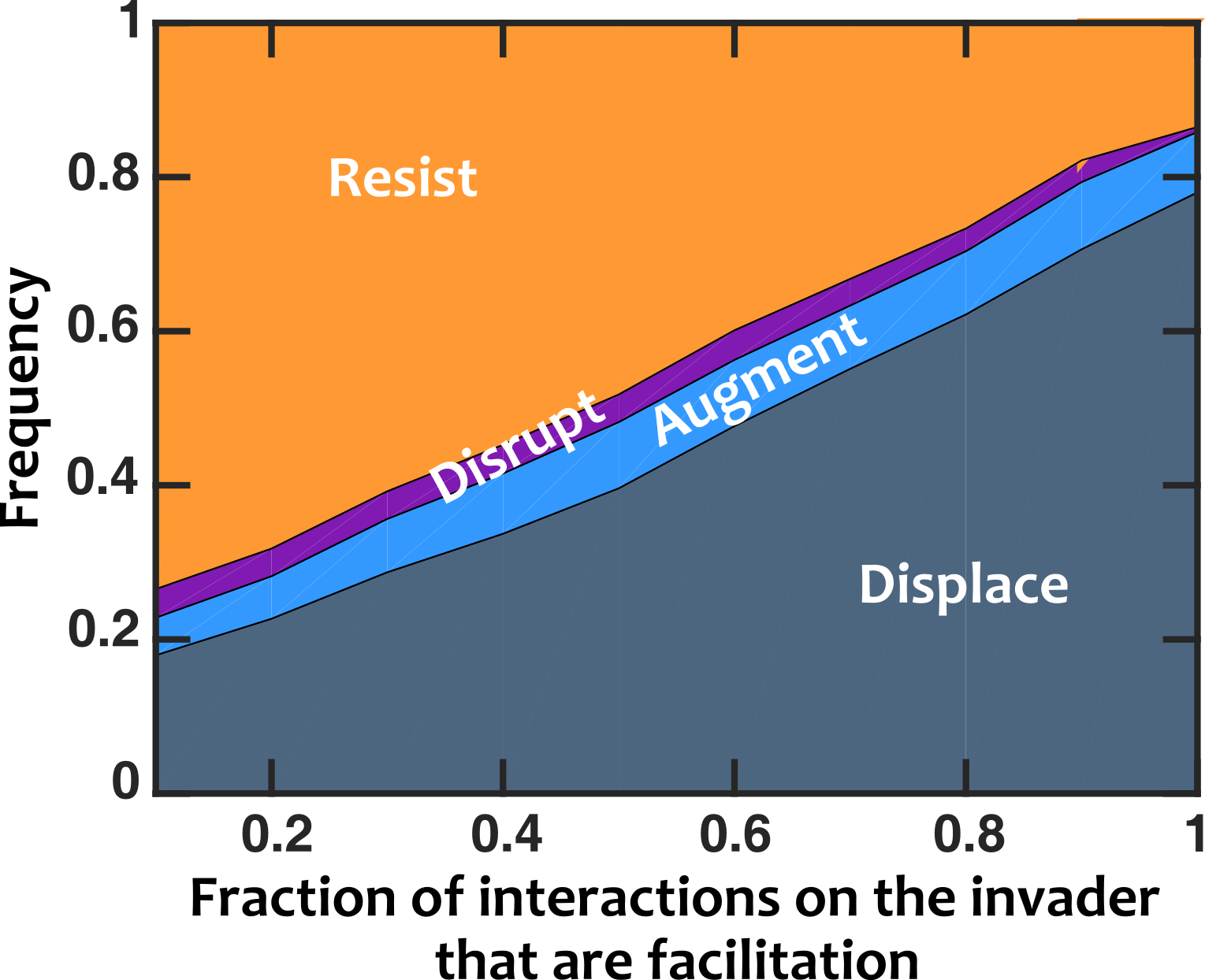

Supplement: S10 Fig — Invasion success drastically increases when we switch the interactions that influence the invader from inhibition to facilitation. Number of instances examined Ns = 1000. Interactions among resident members are equally likely to be facilitative or inhibitory (ffac = 0.5). Normalized basal growth rate of the invader is 1.5. Normalized introduced propagule size is 0.3%. The amount of the limiting resource is set at 106 fmole/ml. (TIF) [file pcbi.1008643.s010.tif]

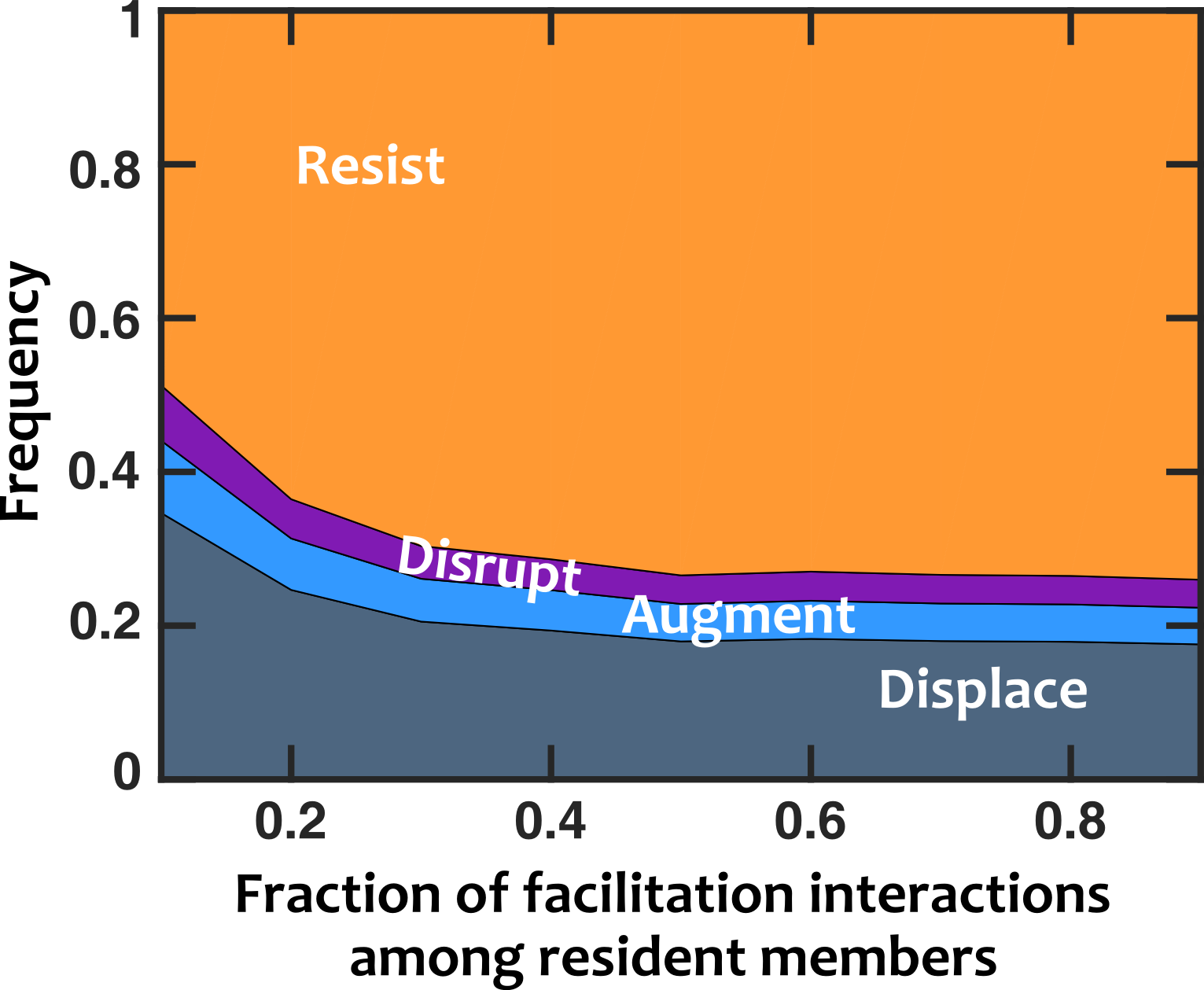

Supplement: S11 Fig — Invasion success decreases when interactions among resident species are predominantly facilitation rather than inhibition. The interactions between resident species and the invader are mostly inhibitory (ffac,inv = 0.1). Normalized basal growth rate of the invader is 1.5 (compared to resident members). Normalized introduced propagule size is 0.3%. The amount of the limiting resource is set at 106 fmole/ml. (TIF) [file pcbi.1008643.s011.tif]
